# Recent Developments on Multi-Functional Metal-Free Mechanochromic Luminescence and Thermally Activated Delayed Fluorescence Organic Materials

**DOI:** 10.3389/fchem.2020.00483

**Published:** 2020-06-30

**Authors:** Debasish Barman, Rajdikshit Gogoi, Kavita Narang, Parameswar Krishnan Iyer

**Affiliations:** ^1^Department of Chemistry, Indian Institute of Technology Guwahati, Guwahati, India; ^2^Centre for Nanotechnology, Indian Institute of Technology Guwahati, Guwahati, India

**Keywords:** delayed fluorescence (DF), phosphorecence, donor - spacer - acceptor structure, organic light emitting diode (OLEDs), mechanochromic fluorescence, aggregation induced emission (AIE), structure property correlation

## Abstract

Metal-free organic compounds with highly ordered π-conjugated twisted skeletons are capable of generating brilliant multi-colored light. Additionally, the co-existence of numerous other multi-functional properties have endowed them with the potential to be a promising class of materials for several electronic and photonic applications and next-generation advanced luminescent material-based devices. This review highlights the recent developments made in this fascinating class of multi-property encompassing materials, involving a highly twisted donor-acceptor based single molecular platform with synchronized photophysical behavior such as thermally activated delayed fluorescence (TADF), mechanoresponsive (MR), room-temperature phosphorescence (RTP), and aggregation induced emission (AIE) with associated unique and inherently manifested structure-property relationship investigations. Furthermore, a brief summary of the optoelectronic behavior of TADF materials are also presented by correlating their performances in the organic light-emitting diodes (OLEDs) and corresponding EL devices. In addition to mechanochromic luminescence (MCL) with TADF behavior, new types of emitters are also being developed, with tunable color changes such as blue-green, yellow-orange, yellow-red, etc., with some emitters crossing the entire visible span to produce white OLEDs. These developments have enriched the library of fascinating organic materials in addition to providing new directions of multifunctional material design for solutions processed OLED and several other advanced devices.

## Introduction

Light is an auspicious source of existence in the living world, especially multicolored bioluminescence from natural light emitting pigments, making the universe more beautiful. Inspired by nature, human beings imagined and developed organic, inorganic, and hybrid materials as a source of chemiluminescence to generate light affordably and created many exciting ways in which to use it, for the benefit of living beings. In view of this, organic light emitting diodes (OLEDs) which are widely exploited in the field of lighting technology and emerging luminescent organic materials, have wielded considerable importance over the past 30 years, to advance the material design and its related functionality, since the first pioneering technology was introduced in 1987 by Tang and VanSlyke (1987). However, in the last 5 years, metal free modern luminescent organic materials with thermally activated delayed fluorescence (TADF) behavior have been gaining prominence as an inevitable class of luminophores, following the invention of first TADF material 4CZIPN by C. Adachi and coworkers in 2012 (Uoyama et al., 2012). Along with notable progress in material synthesis, researchers have devoted persistent efforts to develop underlying fundamental mechanisms and an effective design strategy for multifunctional TADF materials. In particular, TADF materials have a high potential to evolve into versatile applications like flat panel displays and solid-state-lighting (Zhang et al., 2012; Wu et al., 2015; Liu et al., 2018) including photodynamic therapy (Li et al., 2017), sensors (Kinami et al., 2006), security (Yamashita et al., 2005), bioimaging (Hirata and Watanabe, 2006), memory chips (Meher and Iyer, 2018) etc. In the early stages of research, exploration of conventional fluorescence small molecules and polymers had aroused immense research interest. Certain limitations in the device efficiency, however, suffered mainly by non-radiative triplets with a share of ~75% of all excitons, generated directly under electrical excitation with their poor spin-orbit coupling (SOC), weak intersystem crossing (ISC) and solely singlet excitons contribution, restricted their internal quantum efficiency (IQE) to only 25% and external quantum efficiency (EQE) to 5% (Xu et al., 2015). Further, the involvement of Iridium (Ir), Platinum (Pt) and Ruthenium (Ru) based heavy metal complexes greatly enhanced the SOC, activating the phosphorescence emission from triplet state achieving theoretical 100% IQE (Bolton et al., 2011). However, assimilation of highly expensive and non-environmentally friendly precious heavy metals are not preferred in practical applications. In this regard tremendous effort has been made to explore alternative approaches such as hybridized local charge transfer (HLCT) (Li et al., 2014), triplet-triplet annihilation (TTA) (Chou et al., 2014), including TADF, in order to harvest 100% excitons and to overcome the obstacle faced by conventional fast degradable fluorescence/phosphorescence materials (Scholz et al., 2015). Unlike other discoveries, the most successful breakthrough was achieved in the case of TADF materials, which has been realized as a promising material for lighting applications due to the decent EQE and very high photoluminescence quantum yield (PLQY), as compared to inorganic phosphorescence materials (Zhao et al., 2016). In spite of this exciting phenomenon, of additional extreme importance is the discovery of more closely associated functional features accompanying these TADF materials, that include (but are not limited to) aggregation-induced emission (AIE), white-light emission, and multi-color tunability upon severe external stimuli/mechanical force, which creates a mechanochromism property or so-called TADF-mechanophore, which extends their conceivable application manifold (Tonge and Hudson, 2019). The molecule showed multiple color emissions on application of some external stimuli like grinding, recrystallization, and solvent fuming due to the change in its molecular packing mode and intra and inter molecular interactions. Owing to the unique and easy to tune chemical and optical properties, wide range of color switching ability, and huge possibilities on structural flexibility in an economical organic molecular platform, these TADF materials are considered to be a promising avenue for the development of next-generation electronic devices (Das et al., 2019). Moreover, an ever-increasing demand to use them as a solution-processed non-doped fabrication technique gives them an extra edge over other existing methods for solid-state lighting. However, the major issues of efficiency improvement affected by emission quenching in non-doped thin-film state is mainly due to singlet-triplet annihilation (STA), long lifetime triplet-polaron, and triplet-triplet annihilation (TTA) (Ban et al., 2017). Yet, quenching the effect of TADF emitters can be fixed by co-existing with an impressive solid state emission property, called aggregation induced emission (AIE), a condensed state emission behavior that can arrest the rotation or vibration in a highly twisted molecular configuration likewise TADF molecular structure. Further, expanding the functionality and accessibility of the multifunctional-TADF molecules enables it to evolve in new processing techniques such as ink-jet printing, spin coating etc. (Müller et al., 2003; Cho et al., 2014), offering enormous advantages over expensive thermal deposition techniques, and which can be effectively integrated in optoelectronic device fabrication platforms (Albrecht et al., 2017).

Impressively, white-light-generating TADF-single emitters have great potential for use in flat-panel displays and have attracted broad interest in future light sources (Sun et al., 2006; Reineke et al., 2009). Most of the reported organic white-light emitters were accomplished by rational blending of red/green/blue or blue/orange emitters, which covers the complete visible-light emission spectrum region (Shao et al., 2012). Notably, single organic white-light solids were often found by harnessing singlet excitons, like monomer/excimer complex, excited-state intramolecular proton transfer (ESIPT) etc. (Tang et al., 2011). However, white radiative decay from both singlet and triplet excitons have recently been found in TADF molecules (Wu et al., 2019). Moreover, development of single molecule white light emitters (SMWLEs) with multi-functional additional exciting key properties are under investigations due to their superior performance, no phase segregation, no color aging with improved stability, good reproducibility, and simple device fabrication protocols (Sun et al., 2019). In addition, by introducing AIE-ML functionality in white TADF emitters, efficient device results have been realized by overcoming the complex material arrangements and by appropriate doping of materials into the host to achieve high electroluminescence (EL) and high external quantum efficiencies (Zheng et al., 2019b). Simple material based high performance organic white-light emitter fabrication therefore remains a challenge.

## Fundamental Aspects and Working Principles of TADF and MCL Materials

### Approaches to Realize TADF Property

**Figure 1:**
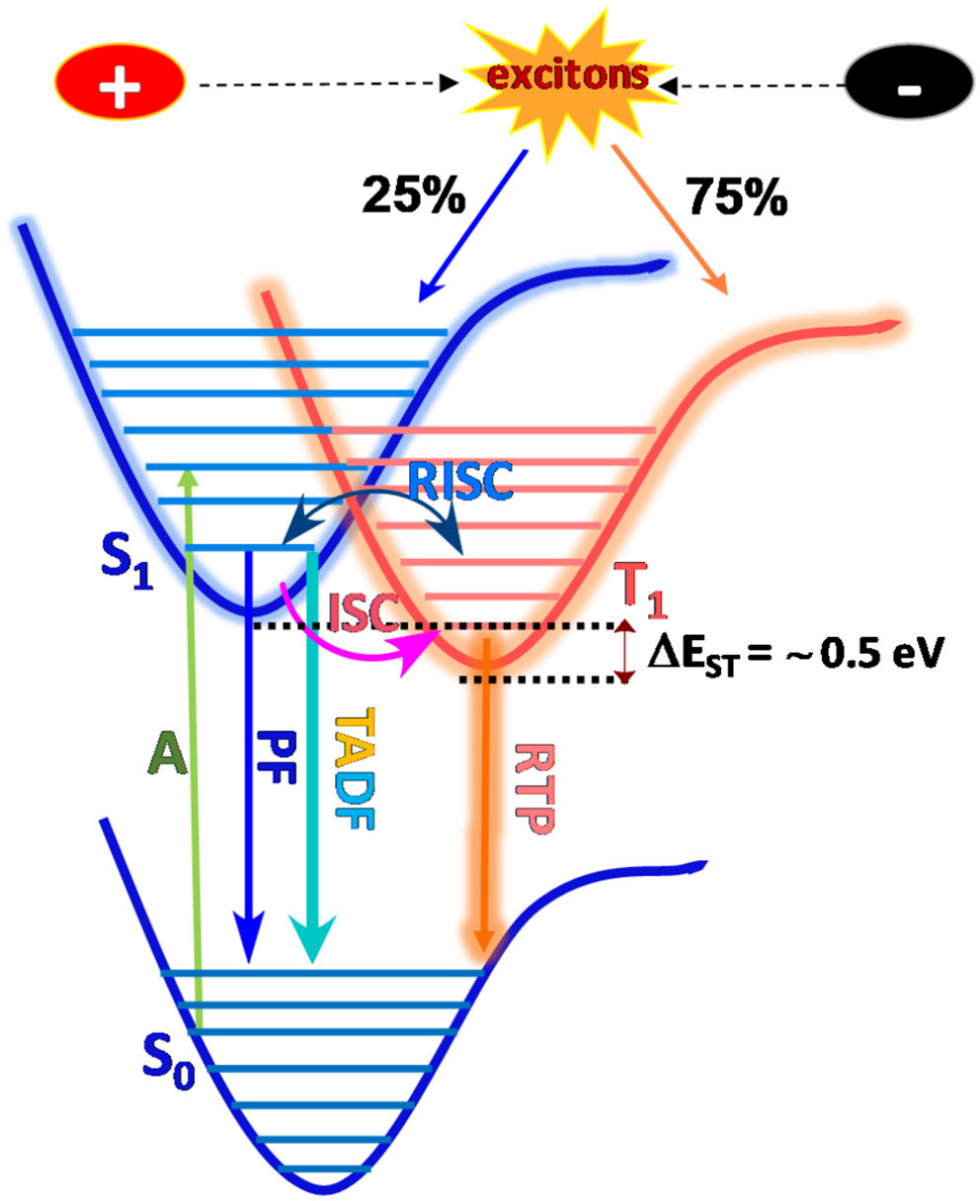
Mechanistic view of Jablonski diagram: multiple emission originates through the radiative decay of the energetically excited state (S_1_) to the ground state (S_0_) and is denoted as prompt fluorescence (PF), TADF, and RTP through the ISC and RISC process.

Organic luminogens with electron donor (D) and electron acceptor (A) containing moieties connected either directly or *via* spacer units in a hindered orientation, are considered to be promising candidates for multifunctional luminescence behavior in terms of high quantum-efficiency, good charge transporting ability in either an intramolecular or intermolecular fashion, which endows them with tunable electronic properties (Tao et al., 2014). Principally, TADF molecules must exhibit strong spin–orbit coupling which is generally induced by rapid conversion of the singlet and triplet manifolds, without the use of heavy elements and simultaneously without a small energy gap between low lying singlet and triplet states. Consequently to achieve high quantum-efficiency, the molecule should exhibit faster radiative decay *via* charge transfer states, which minimizes the exchange energy and therefore the singlet-triplet gap (Penfold et al., 2018). In particular, most efficient TADF emitters are based on covalently bonded donors and acceptor conjugated backbones, with features of through-bond charge transfer (TBCT) which is crucial to realize efficient TADF (Ahn et al., 2019), while few of them report to date are through-space and polymers (Tsujimoto et al., 2017). Nonetheless, a strong electron coupling between donors and acceptors mediated by covalent bonds enables the display of large oscillator strength and high PLQY (Nobuyasu et al., 2019). However, due to the lack of universal design methodologies and principles, the development of organic luminogens with multicolor and white-light emissions are predominantly still under investigation. Generally, these types of materials possess numerous versatile photophysical phenomenon brought about by radiative emission, generally mediated through intramolecular charge transfer (ICT), vibrational relaxation, singlet-triplet intersystem crossing (ISC), internal conversion (IC), vibrational relaxation, etc., resulting in either fluorescence and phosphorescence (Itoh, 2012). Emission and relaxation processes are thus carefully investigated by manipulating excited states for donor-acceptor structural configurations (Berberan-Santos and Garcia, 1996), where emission depends on their different excited state's energy levels and probable excitons transition over these states. Therefore, by tuning the excitons of electronically excited states with effective transitions, different and distinct excited energy levels with effective transitions evolved through which photophysical processes can be precisely controlled by means of lifetime and quantum yield (Wu et al., 2018) (summarized in Jablonski diagram Figure 1).

According to the El Sayed rule, the photophysical processes under photonic/electronic irradiation of luminogens typically produce 25% of singlet and 75% of triplet excitons, which can travel from excited singlet (S_1_) to ground state (S_0_) (i.e., fluorescence), which radiates instantly with the lifetime of a nanosecond (ns), and where the transition from triplet excited state (T_1_) leads to non-radiative decay due to spin being forbidden (Lower and El-Sayed, 1966). However, TADF is a delayed emission that originates from designing an aromatic compound with bulky steric hindrance and which is expected to resolve the issue of sinking triplet excitons. Surprisingly, triplet excitons can be upconverted into singlet, giving it additional enhanced fluorescence and a lifetime of up to several microseconds. Moreover, the reverse intersystem crossing (RISC) process was efficiently facilitated through a very low ΔE_ST_ (energy gap between S_1_ and T_1_) value (~0.3 eV, Sun et al., 2014; Wong and Zysman-Colman, 2017; Wei et al., 2019) which is correlated with the molecular structure of the TADF emitter, as it is proportionate to the exchange integral (J) as given in equation 1 & 2 (Shizu et al., 2015). Importantly, exchange integral, J depends on the electron density overlap between the highest occupied molecular orbital (HOMO) and lowest unoccupied molecular orbital (LUMO), assuming that the S_1_ and T_1_ states are influenced by HOMO to LUMO transitions and a minimum energy difference is derived/devised from a well-separated HOMO/LUMO in a highly twisted aromatic D-A skeleton (Sun et al., 2014).

(1) $$\begin{array}{r} \Delta {\text{E}}_{\text{ST}}={\text{E}}_{\text{S}}-{\text{E}}_{\text{T}}=2\text{J} \end{array}$$

(2) $$\begin{array}{r} \text{J}=\;\iint \emptyset_{\text{HOMO}}\left({\text{r}}_{1}\right)\emptyset_{\text{LUMO}}\left({\text{r}}_{2}\right)\frac{1}{\left|{\text{r}}_{2}-\;{\text{r}}_{1}\right|}\emptyset_{\text{HOMO}}\left({\text{r}}_{2}\right) \end{array}$$

$$\begin{array}{l} \emptyset_{\text{LUMO}}\left({\text{r}}_{1}\right)\text{d}{\text{r}}_{1\text{d}}{\text{r}}_{2} \end{array}$$

Where, ∅_HOMO_ and ∅_LUMO_ are termed as spatial distributions of the HOMO and the LUMO, and r_1_ r_2_ are position vectors, respectively (Moral et al., 2015). It obeys the very small overlap between the HOMO and the LUMO, hence, decreases the exchange integral (J) and ΔE_ST_ value. Subsequently, if the ΔE_ST_ is sufficiently moderate, or is in the presence of a non-metallic heavy atom, carbonyl, thioesters, or when assisted by polymer host matrix, both SOC and faster ISC processes were favored, thereby leading to triplet emission at room temperature, called room temperature phosphorescence (RTP), which has a lifetime span of microseconds to seconds (Chen and Liu, 2019). Therefore, the primary goal is to utilize triplet excitons to achieve 100% internal quantum efficiency (IQE) by utilizing the concept of either TADF or RTP. Moreover, accurate quantum yields of fluorescent (φ_*F*_) and TADF (φ_*TADF*_) materials with the rate constants of fluorescent (*k*_*F*_), internal conversion (*k*_*IC*_), TADF (*k*_*TADF*_), intersystem crossing (*k*_*ISC*_), reverse intersystem crossing (*k*_*RISC*_), and efficiency of ISC (φ_*ISC*_), RISC (φ_*RISC*_) can be obtained from formulas 3–8 in the solid state (Lin et al., 2016). By following the aforementioned equation, ΔE_ST_ can also be calculated from formula-9, where R and T signify the ideal gas constants, respectively.

(3) $$\begin{array}{r} \varphi =\;\frac{\kappa_{F}}{\left(k_{F}+k_{IC}\right)} \end{array}$$

(4) $$\begin{array}{r} \varphi_{ISC}=\;\frac{\kappa_{F}}{\left(k_{F}+k_{IC}+k_{ISC}\right)}=\;\kappa_{F}\tau_{F} \end{array}$$

(5) $$\begin{array}{r} \varphi_{ISC}=\;\frac{\kappa_{ISC}}{\left(k_{F}+k_{IC}+k_{ISC}\right)}=\;\kappa_{F}\tau_{F} \end{array}$$

(6) $$\begin{array}{r} \frac{\varphi_{TADF}}{\varphi_{F}}=\frac{\left({\;\varphi }_{ISC}\varphi_{RISC\;}\right)}{\left(1-k_{ISC}k_{RISC}\right)} \end{array}$$

(7) $$\begin{array}{r} k_{TADF}=\;\frac{\varphi_{TADF}}{\left(\varphi_{ISC}\tau_{TADF}\right)} \end{array}$$

(8) $$\begin{array}{r} k_{RISC}=\;\frac{{k_{F}k_{TADF}\varphi }_{TADF}}{\left(k_{ISC}\varphi_{F}\right)} \end{array}$$

(9) $$k_{TADF}=\frac{1}{\left(3k_{F}\exp(-\Delta E_{ST}/RT\right)}$$

### Approaches to Realize MCL Properties

Mechanochromic luminescence (MCL)/electroluminescence (EL) properties are switchable optical processes, which involve many other types of emissions in a viable functional molecule that co-exists with TADF and RTP properties. Furthermore, it remains extremely challenging to design a molecule with different optical properties in a single molecular platform and has aroused a broad potential utility and inherent luminescence mechanism to develop a new class of multi-functional materials. Considering this view, the phenomenon of altering the material properties by external factors such as light, heat, pH, pressure, magnetic, or by electric field of stimuli, is known as “Piezo luminescence” and other terms like mechanofluorochromism, mechanochromic luminescence, and piezochromism are also often used in the literature for the interchangeable properties (Sagara et al., 2007). However, the exact mechanism of MCL is not deliberated precisely, although many strategies that have been investigated, provide certain requirements to design molecules with MCL/MR properties. Yet, the reported MR materials with thermodynamically metastable states which have varying supramolecular assemblies or high order crystallinity, including the possibilities of conformational change, excimer formation, excitons coupling, and other intermolecular interactions such as hydrogen bonding, ionic interactions, π-π stacking, Van der-Waals forces, etc., are found to exist (Kasha et al., 1965; Birks, 1975; Schmidbaur and Schier, 2008). Furthermore, aggregation induced emission (AIE) has also been included as a fascinating condensed state property that meets a few requirements such as TADF and MCL/MR, enabling comprehensive and versatile explorations such as security, sensors, data-storage and many other applications (Sagara et al., 2016).

Therefore, TADF materials with multicolor and multi-functional inclusive properties and with a significant AIE-ML field of research, has been in the spotlight recently. Additionally, single molecule white-emissive TADF material with bicolor ML strategies and molecular systems are highly desirable and have rarely been reported so far.

## Recent Progress on Multi-Functional TADF Emitters

### Dual-Color Emission Switching TADF-MCL/ECL

Metal free organic compounds which exhibit tunable emission and TADF, are commonly found in donor-acceptor type molecular systems which possess strong charge transfer ability, including well-separated HOMO/LUMO, resulting in a very low ΔE_ST_ value (~0.3 eV), thereby facilitating the RISC process effectively and exhibiting the TADF property with high quantum efficiency (Data and Takeda, 2019). D-A materials therefore display reversible and distinct emission in response to external stimuli such as rubbing, grinding, shearing, pressing, temperature, electric field, and vapor and have found multiple applications in organic electronics, sensors, probes, security inks, and many more (Chi et al., 2012). This type of MCL behavior is due to the reversible changes in chemical structures or reformed conformations that do not necessarily, at all times, require breaking chemical bonds, or in physical structures with their different packing modes in the same molecule but thermodynamically stable and metastable states mostly enabling tunable emission. Multi-color changing MCL materials are therefore promising tools toward sensitive sensing of physical environments such as temperature, pressure, and pH and hence they have emerged as a fascinating area of research within the last 5 years.

**Figure 2:**
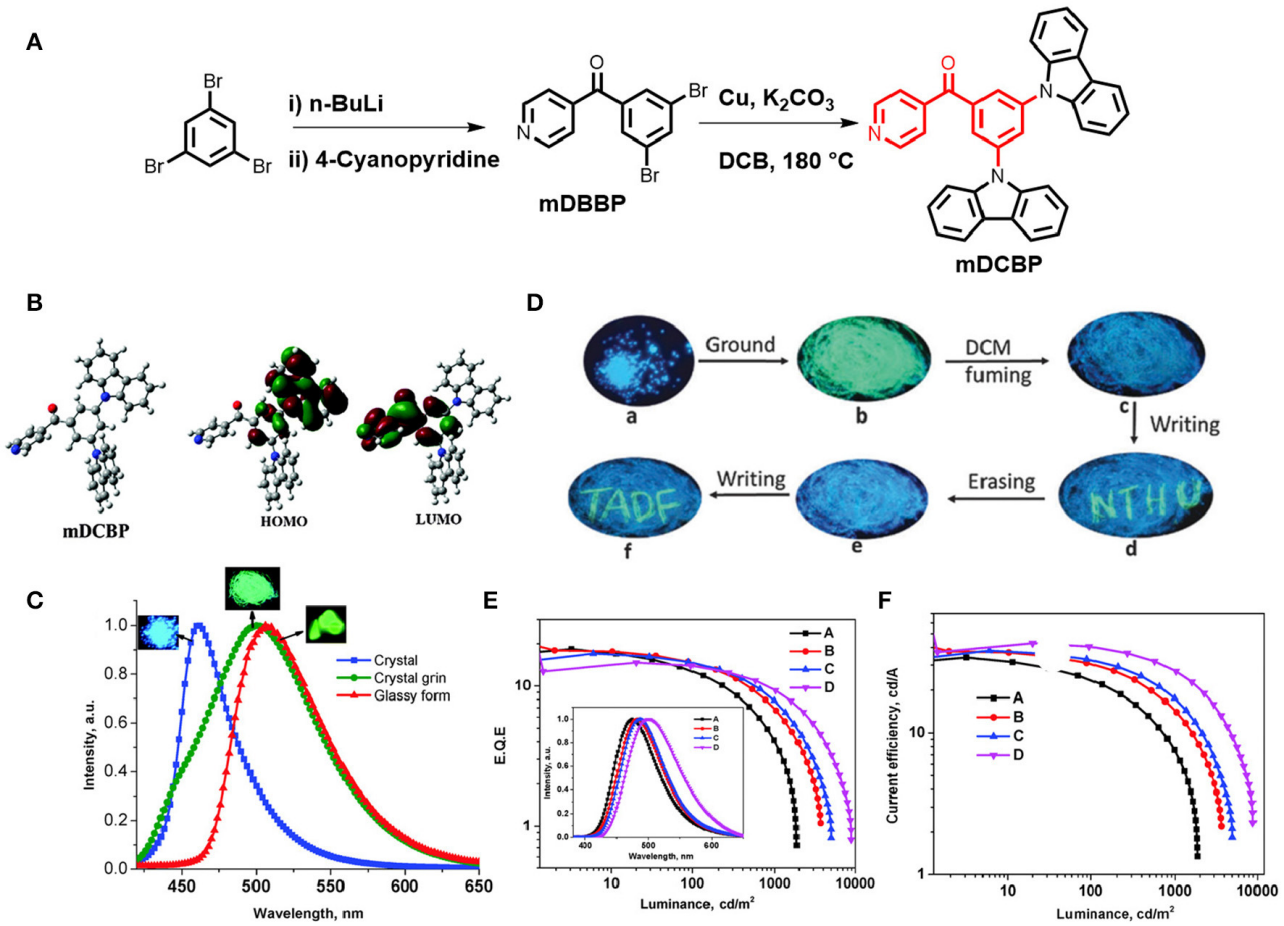
**(A)** Chemical structure of mDCBP and its synthetic scheme. **(B)** Optimized molecular configuration of mDCBP and calculated HOMO-LUMO with spatial distribution. **(C)** Emission spectra of mDCBP in its various forms (crystalline, amorphous and glassy); insets are the respective images under UV-irradiation. **(D)** MCL behavior of mDCBP in response to various external mechanical stimuli. **(E)** External quantum yields of the fabricated devices **(A–D)**; insets are the EL spectra of the devices. **(F)** Current efficiency vs. luminescence plot. Reproduced with permission from Rajamalli et al. (2016). Copyright © The Royal Society of Chemistry.

A TADF emitter (3,5-di(9H-carbazol-9-yl)phenyl)(pyridin-4-yl)methanone (*m*DCBP) comprising two meta carbazolyl units as electron donating moieties and a benzoylpyridine core as an electron accepting moiety was developed by Rajamalli et al. (2016) (Figure 2). The D-A type molecular system was clearly revealed by the DFT study, where, HOMO of the molecule is mostly localized over the donor moiety, whereas the LUMO is localized over the acceptor unit resulting in a very small Δ**E**_**ST**_ value of 0.06 eV. This small Δ**E**_**ST**_ value facilitates the RISC process to exhibit TADF property. The molecule exhibited highly enhanced emission in solid state, in addition to a reversible, externally tunable emission (blue emission in the crystalline form and green emission in the amorphous form) response, investigated by alternating applied mechanical stimuli and solvent fuming, (detail theoretical and photophysical results for *m*DCBP-emitters are summarized in Table 1). The different colored emission by the same molecule was due to the change in its molecular packing mode under a different mechanical force and external stimuli. For example, the emission maximum was red shifted from 460 to 500 nm after grinding of mDCBP. The red shifting is likely due to the enhancement in the intermolecular interactions triggered by the applied external pressure. The TADF properties were clearly revealed by the transient PL measurements and the lifetime of the delayed component was found to be 0.02 μs, and the excited state lifetime was lower than the phosphorescent complexes with iridium. TADF-based blue and green OLEDs have been fabricated using *m*DCBP as the emissive layer, where maximum EQE were found to be 18.4 to 14.7%, respectively (detailed device configuration and performances are summarized in Table 2).

**Table 1:** Computational and Photophysical characterizations of TADF-MCL emitters.

**Emitter**	**HOMO (eV)**	**LUMO (eV)**	**Osc. Strength(*f*)**	**Theo. ΔE_**ST**_ (eV)**	**Exp. ΔE_**ST**_ (eV)**	**λ_max, _ abs (nm)**	**λ_max, _ PL (nm)**	**^a^_*τ*P_(ns)**	**^b^_*τ*T_(μs)**	**^c^ϕ [%]**	**^d^BSλ_max, _ PL (nm)**	**^e^ASλ_max, _ PL (nm)**	**^f^T_d_/T_g_ (^°^C)**	**References**
mDCBP	5.72	2.72	-	0.15	0.06	334, 372	467	6.2	0.2	90	460	500	394/105	Rajamalli et al., 2016
T_2_	−4.97	−2.34	-	0.18	**-**	-	-	4.23	78.5	33.6	433		382/95	Ganesan et al., 2017
2CzPN	-	-	-	-	-	374	533	33.3	20	11				Ishimatsu et al., 2014
1_R	−5.31	−2.85			0.08	416	657	57.8	1.1	7	568 (1_Y), 640 (1_O)	673 (1_R)		Okazaki et al., 2017
5TzPm-PXZ	−5.16	−3	-	0.21	0.10	317, 421	567	-	2.9	64	583	599	471/107	Zeng et al., 2018
MeO2Qx	−5.75	−2.90	-	-	0.14	437	582	-	-	-	534	586	365/110	Pashazadeh et al., 2018b
DMAC-CNQ (Y_crystal)	−5.41	−2.94	-	0.04	-	347, 453	572	28	9.6	34	539	610,576		Zheng et al., 2019a
FDMAC-CNQ (Y_crystal)	−5.49	−3.02	-	0.03	-	343, 442	552	7.3	5.1	13	603	620,561		Zheng et al., 2019a
PTZ-AQ (R-crystal)	–	-	0.0137 (S_1_), 0.0594 (S_2_)	0.51	0.01	239, 299, 443	606	-	275.6	84	606	~545	-	Huang et al., 2018
TATC-BP	−5.30	−2.83	-	0.08	0.125	324	524	53.5	0.94	22	483	542	>400/ >95	Chen et al., 2018
QBP-DMAC	−5.28	−2.83	-	0.40	0.33	342	508	15, 13 (doped)	1.57, 1870(d-oped)	78	463	525	427/137	Zheng et al., 2019b
DPPZS–DBPHZ	-	-	0.652	0.30	-	397, 420	484	-	-	34	497	534	-	Takeda et al., 2018
OIDBQx	−5.26	−2.75	-	-	0.49	287, 315, 411	534	1.7 ± 0.2	-	31	494	522	398/146	Pashazadeh et al., 2018a
PTZ-DBPHZ (1_R)	-	-	-	-	-	-	-	38.42	0.98	66	568 (1_Y)	673 (1_R), 640 (1_O)	-	Data et al., 2019
Mono-DMACDPS	−5.30	−2.22	-	0.014	0.09	362	471	-	3.8	39	-	**~**470	298/69	Zhan et al., 2019
3-DPH-XO (crystal-C)	-	-	-	-	0.02	280, 370	540	8.61	221	44	-	-	-	Zhang et al., 2017

a *Prompt fluorescence lifetime (τ_P_)*,

b *TADF lifetime (τ_T_)*,

c *photoluminescence quantum yield (ϕ)*,

d *before stimuli (BS)*,

e *after stimuli (AS)*,

e *thermal decomposition temperature (Td)/glass transition (Tg) temperature*.

***N.B**. All photophysical studies in toluene Rajamalli et al., 2016; Ganesan et al., 2017; all photophysical studied on neat film Chen et al., 2018; Zhan et al., 2019; all photophysical studies in DCM Takeda et al., 2018; all photophysical studies in their respective solid states/crystalline states (Zhang et al., 2017; Huang et al., 2018; Data et al., 2019; Zheng et al., 2019a); only UV/PL in toluene (Okazaki et al., 2017; Pashazadeh et al., 2018b; Zeng et al., 2018; Zheng et al., 2019b); only UV/PL in DCM Ishimatsu et al., 2014; Pashazadeh et al., 2018a; life time study on thin film (CBP host) Zeng et al., 2018; Zheng et al., 2019b; life time study on thin film (zeonex host) (Pashazadeh et al., 2018a,b)*.

**Table 2:** TADF-OLED device configurations and related EL-device performances.

**Emitter**	**Device structure**	**Turn on Voltage (V)**	**CIE values**	**^a^EQE (%)**	**Brightness (Cd/m^**2**^)**	**^b^CE (Cd/A)**	**^c^LE (lm/W)**	**Ref**.
mDCBP	ITO/NPB /mCP /DPEPO:mDCBP /PPT/TmPyPb /LiF /Al	3.6	(0.16; 0.25)	18.4	1,870	34.0	26.5	Rajamalli et al., 2016
T2	ITO/TAPC/mCP:T2/DPEPO:T2/TmPyPB/LiF/Al	3.0	(0.20, 0.39)	14.2	7,385	34.2	29.8	Ishimatsu et al., 2014
1	ITO/NPB/CBP:1/TPBi/LiF/Al	-	-	16.8	≥25,000	19.6	-	Okazaki et al., 2017
5,7TzPmPXZ	ITO/(PEDOT:P-SS)/ CBP:5,7TzPmPXZ / (TmPyPB)/Liq/Al	-	(0.43, 0.53)	14.3	12,210	41.9	-	Zeng et al., 2018
MeO2Qx	ITO/HIL)/PVK: PBD-MeO2QX/TPBi/LiF/Al	5.0	(0.41, 0.53)	10.9	16,760			Pashazadeh et al., 2018b
TATC-BP	ITO/PEDOT:PSS/TATC-BP/TmPyPB/LiF/Al	2.6	(0.41, 0.54)	5.9	-	17.8	20.0	Chen et al., 2018
TATC-BP	ITO/PEDOT:PSS/TATC-BP (30 wt%) :H2/TmPyPB/LiF/Al	2.8	(0.37, 0.53)	15.9	-	48.1	47.8	Chen et al., 2018
QBP-DMAC	ITO/ TAPC/ TCTA/CBP: QBP-DMAC/ TmPyPB/LiF/Al	3.6	(0.30, 0.53)	18.8	2,264	56.8	55.8	Zheng et al., 2019b
1	ITO/HIL PEDOT:PSS /CBP:1–DEV_EVP_/TPBi/LiF/Al	2.7		16	28,642			Zheng et al., 2019b

a *External quantum efficiency (EQE)*,

b *current efficiency (CE)*,

c *luminous efficiency (LE)*.

**Figure 3:**
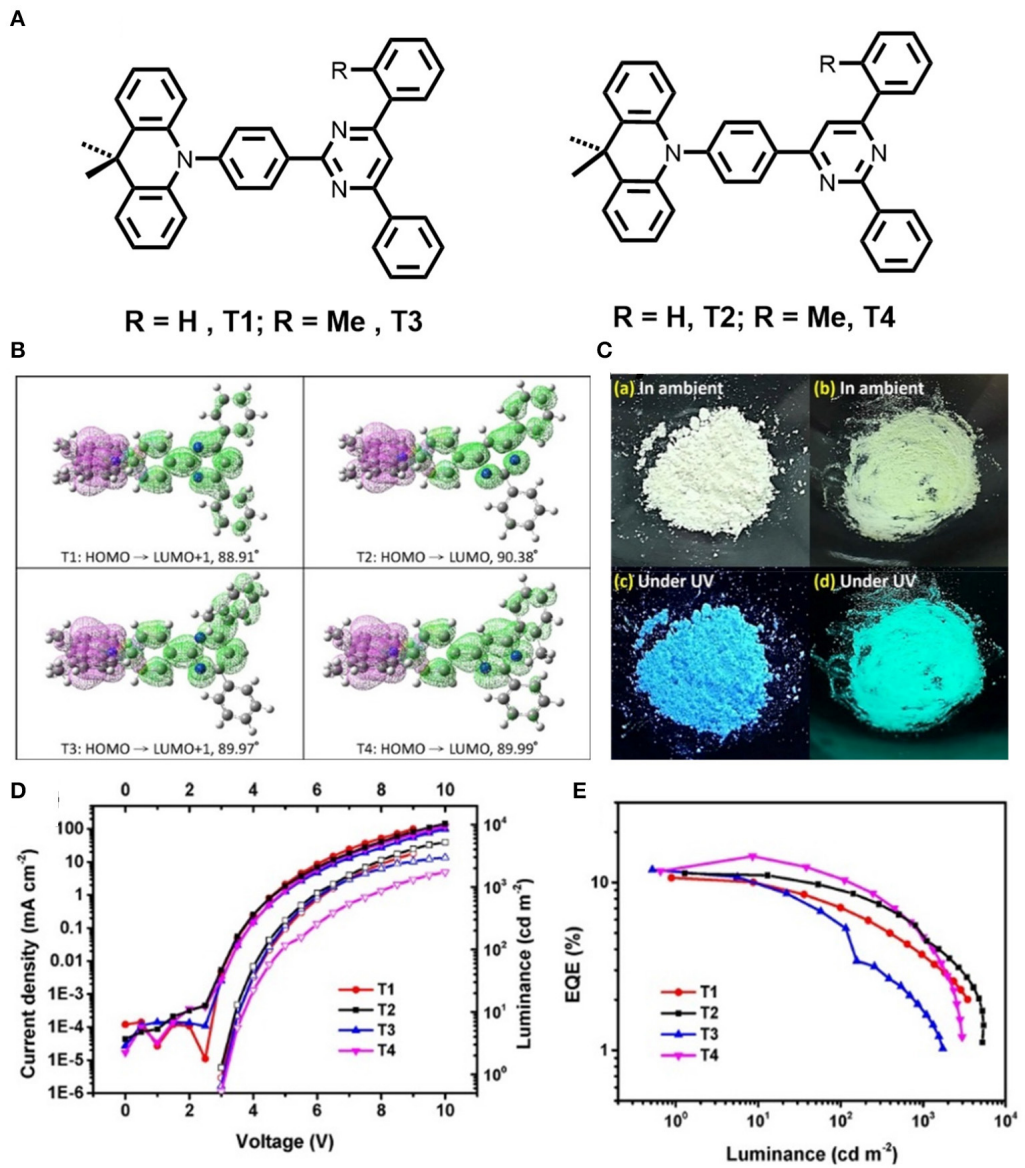
**(A)** Chemical structures of pyrimidine-acceptor core based TADF molecules. **(B)** Frontier molecular orbitals (HOMOs and LUMOs) related to the minimum optical transitions with calculated dihedral angles between the planar dimethyl acridine to the central phenylene substituent are 88.91° and 90.38° 89.97°, and 89.99° for T1-T4 in DCM, respectively. **(C)** Photographs of the ambient and luminescence materials under UV-lamp; color changes of T2 in response to mechanical grinding. **(D)** Current density (J) -voltage (V)-luminance, and **(E)** EQE-luminance plots of the white OLEDs. Reproduced with permission from Ganesan et al. (2017). Copyright ©WILEY-VCH Verlag GmbH andamp; Co. KGaA, Weinheim.

In previous work pyridine moiety was found to be an important role to realize both TADF and MCL: Later, pyrimidine moiety was introduced by Ganesan et al. (2017) and a set of four new molecules, T1-T4, were synthesized, where dimethyl acridine was used as the electron donor and pyrimidine as an electron acceptor (shown in Figure 3). Pyrimidine comprises less stabilized unoccupied π orbitals, which facilitates further functionalization. Notably, the subsequent D-A functional molecules exhibited significant TADF characteristics in solution. Theoretical studies reveal that the asymmetric arrangement of nitrogen atoms in pyrimidine for T2 exhibited the smallest ΔE_ST_ (0.18 eV), thereby a high possibility of thermal up-conversion (T_1_ to S_1_) and hence the high TADF efficiency. Additionally, the materials exhibited reversible dual emission mechanochromism, which leads to evidence of the existence of two types of molecular packing that give distinct emission colors. The photophysical studies of the materials further confirmed that they undergo both prompt fluorescence and TADF (detailed computational and photophysical properties for T2 is given in Table 1). Based on these emitters, OLEDs have been fabricated by employing T2 as an emissive layer, which leads to blue emission with an excellent EQE of 14.2% (Table 2).

**Figure 4:**
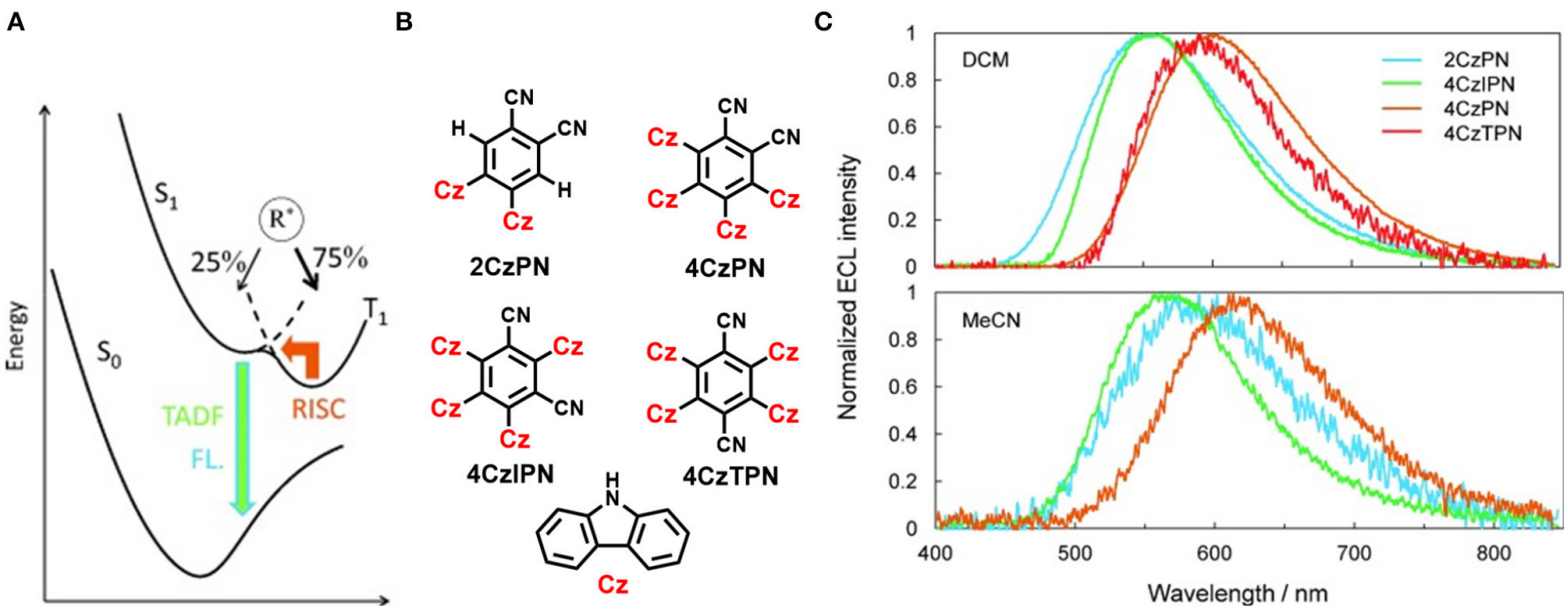
**(A)** Electronic transitions representation **(B,C)** Four TADF molecules and their molecular structure normalized electrogenerated chemiluminescence (ECL) spectra of TADF molecules in DCM (top) and MeCN (bottom). Reproduced with permission from Ishimatsu et al. (2014). Copyright © WILEY-VCH Verlag GmbH andamp; Co. KGaA, Weinheim.

Notably, a series of D-A type TADF molecules (2CzPN, 4CzPN, 4CzIPN, and 4CzTPN) have been reported by Ishimatsu et al. (2014) where carbazolyl is chosen as the electron donor and dicyanobenzene units are used as the acceptor (shown in Figure 4). Ion–ion annihilation of a radical cation and anion, resulted from an electrochemical reaction that produces an excited state species (R^*^), which leads to an emission termed electrogenerated chemiluminescence (ECL). Fascinatingly, these molecules exhibited electrogenerated chemiluminescence (ECL) of intense green to red property and an ECL efficiency of up to 50% was achieved, which is comparable with the corresponding PLQY by applying a square-wave voltage. For common fluorescent molecules there is no contribution of the T_1_ state toward ECL due to the non-radiative T_1_ to S_0_ transition, and, hence, the maximum efficiency of ECL is estimated to be only 25%. Thus, this 50% ECL efficiency indicates that the thermally activated spin up-conversion is very convenient for this particular ECL.

### Multi-Color and Multi-Functional Emission Switching From Crystalline TADF-MCL

In general donor-acceptor systems are very flexible to show dual emission switching under external stimuli. However, it is very challenging to obtain multicolor emission switching from a single molecular system unless it bears a non-planar geometry, either of the donor or acceptor group. Interestingly, most of the materials in this category are very crystalline in nature enabling it to exhibit multi-color emission and multiple forms of crystals, through a simple tuning of the crystal growth environment and non-covalent interactions, to address the structure-property relationship with the TADF phenomenon. In this regard, some of the emitters showed room temperature phosphorescence (RTP), a rare class of photophysical properties of purely organic compounds. Impressively, all the non-radiative excitons can be easily harvested, and device efficiency could be improved by this additional functionality of TADF-MCL emitters.

**Figure 5:**
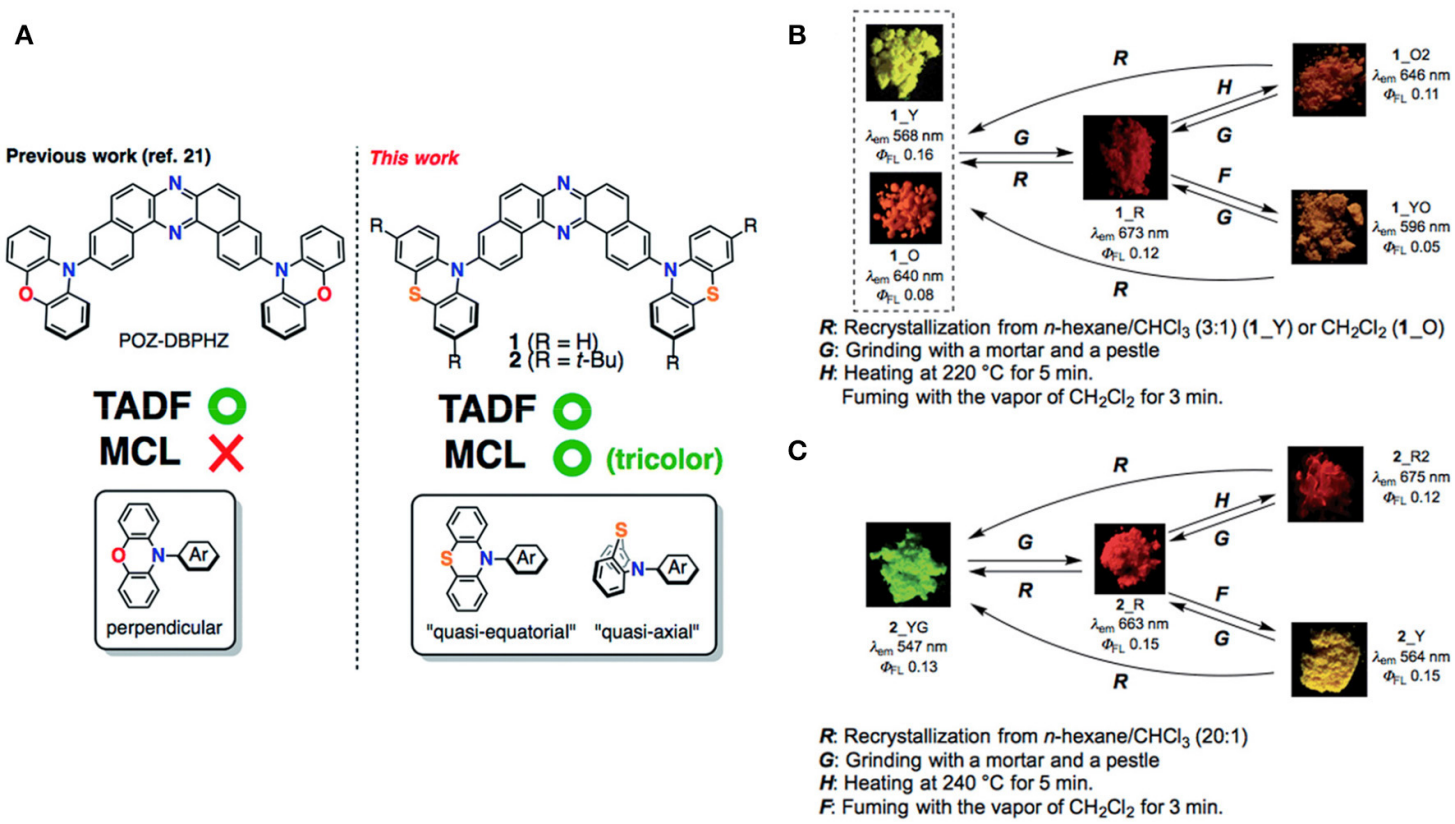
**(A)** Molecular configurations of DBHZ-cored D-A-D triads. **(B,C)** Schematic representation of mechanoluminescence property of 1 and 2, respectively, under a 365 nm UV lamp. Reproduced with permission from Okazaki et al. (2017). Copyright © The Royal Society of Chemistry.

On the aforementioned view, Okazaki et al. (2017) developed novel donor-acceptor-donor (D-A-D) type U-shaped π-conjugated multifunctional molecules, where the dibenzo[*a,j*]phenazine (DBPHZ) unit was chosen as an acceptor and phenothiazines (PTZ) as donors (shown in Figure 5). These molecules showed impressive tricolor-MCL properties, regulated by “two conformation switchable” PTZ units. The D-A-D triad, which is perpendicularly twisted, facilitates efficient intramolecular charge-transfer (ICT) and sufficiently small ΔE_ST_ around 20 meV, which triggers the efficient RISC to achieve efficient TADF characteristics. The related photophysical and theoretical results are given in Table 1 for red emitter1 (R_1). Time-resolved photophysical measurements of the compounds further confirmed that they underwent TADF emission. Notably, in the D-A-D triad, the bowl-shaped structure of PTZ molecules would allow it to exist as two individual conformers, one is quasi-axial and another quasi-equatorial. These “two conformation switchable” PTZ units under application of grinding, heating, fuming, and recrystallization, exhibit different packing modes leading to impressive multicolor emission. Additionally, upon recrystallization and depending on different packing modes and interactions, compound **1** exhibited two polymorphic structures 1_Y (yellow) and 1_O (orange). However, POZ-DBPHZ (oxygen instead of sulfur in the donor), is devoid of MCL and polymorphism due to the planar oxygen atom containing donor. Moreover, an OLED device was fabricated using these TADF emitters and very impressive external quantum efficiencies (EQEs) up to 16.8% was observed, exceeding that of the theoretical data of orthodox fluorescent emitters.

**Figure 6:**
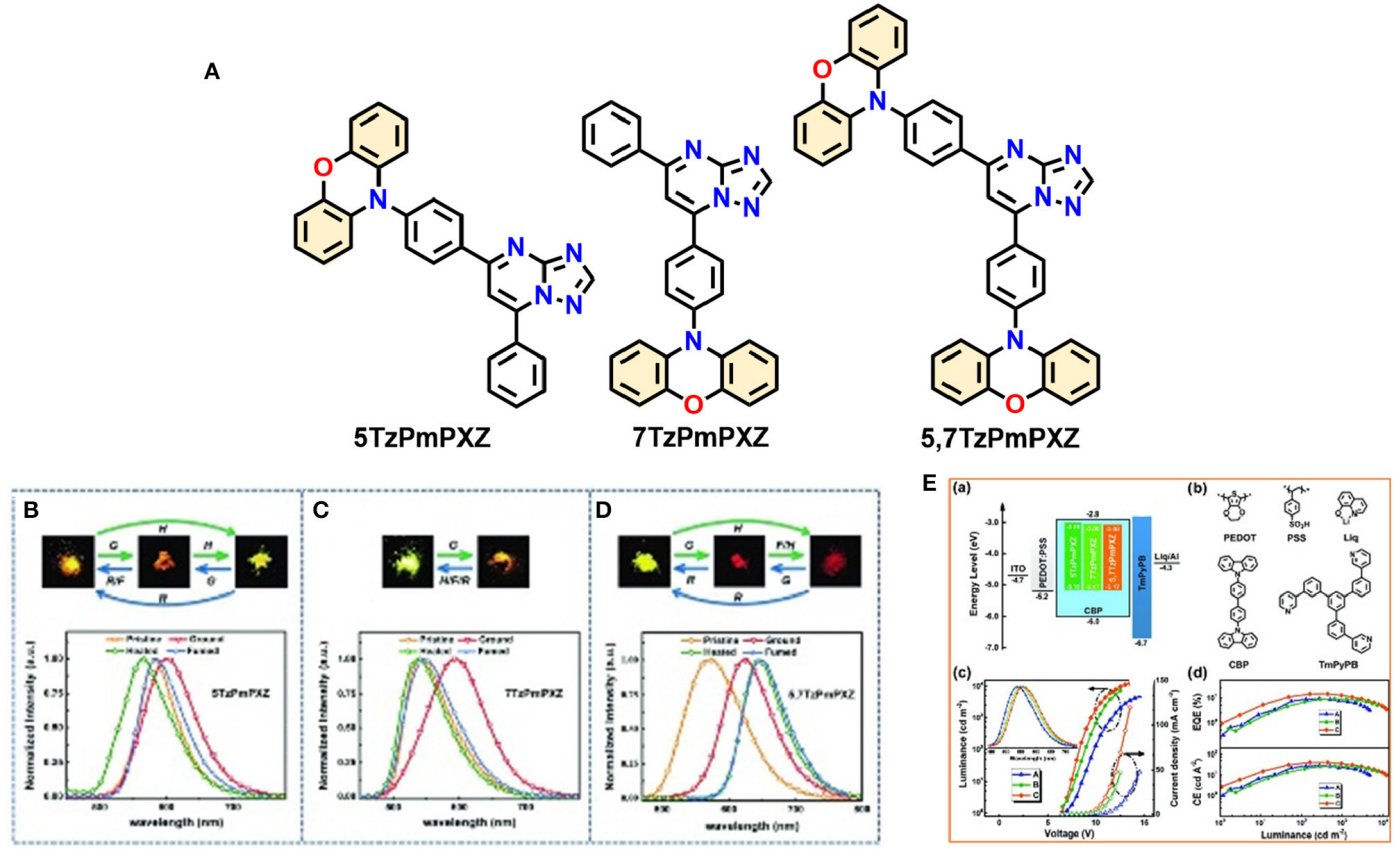
**(A)** Molecular structures of studied compounds. Photos and PL spectra of **(B)** 5TzPmPXZ, **(C)** 7TzPmPXZ, and **(D)** 5,7TzPmPXZ in response to external stimuli. Under 365nm UV irradiation (G: grinding with a mortar and a pestle; H: heating at 150°C for 5TzPmPXZ and 7TzPmPXZ, 200°C for 5,7TzPmPXZ; F: fuming with CH_2_Cl_2_ vapor; R: recrystallization from n-hexane/CHCl_3_). **(E)** OLED performances: (a) the energy level diagrams for the devices A, B, and C, (b) chemical structures of HTL-PEDOT:PSS, CIL-Liq, Host-CBP, and ETL-TmPyPB. (c) J-V-L curves for devices A, B, and C (inset: the normalized EL spectra of devices). (d) EQE and LE vs. luminance curves for devices A, B, and C. Reproduced with permission from Zeng et al. (2018). Copyright © WILEY-VCH Verlag GmbH andamp; Co. KGaA, Weinheim.

Lateron Zeng et al. (2018) reported three multifunctional organic emitters (5TzPmPXZ, 7TzPmPXZ, and 5,7TzPmPXZ) comprising PXZ-moiety integrated with electron-acceptor [1,2,4]triazolo[1,5-a]pyrimidine (TzPm) units, that exhibited TADF with excellent photoluminescence quantum yields (PLQYs) of 49–66%, along with MCL properties caused by transitions between microcrystalline and amorphous phases, in response to external mechanical stimuli. These D-A functional molecules exhibited small ΔE_ST_ (0.06–0.10 eV) including good spatial separation between the HOMO and the LUMO, which is greatly encouraged by the large torsion angle between the donor and acceptor moieties (74.68 to 88.17°) resulting from the steric hindrance between the donor units and the neighboring phenyl ring, susceptible to RISC as well as TADF. Interestingly, 7TzPmPXZ exhibits reversible bicolor-tuning MCL, whereas the 5TzPmPXZ and 5,7TzPmPXZ exhibited tricolor-switching behavior in their solid-state (shown in Figure 6 and detailed TADF-MCL properties for 5TzPmPXZ are discussed in Table 1). Reversible phase transitions between amorphous and microcrystalline states during and after the application of external mechanical force are responsible for multi-color changes. Furthermore, solution-processed OLEDs were fabricated by employing the synthesized compounds in the emissive layer to explore their EL properties. A maximum EQE of 14.3% was achieved for a 5,7-TzPmPXZ-based device which is summarized in Table 2.

**Figure 7:**
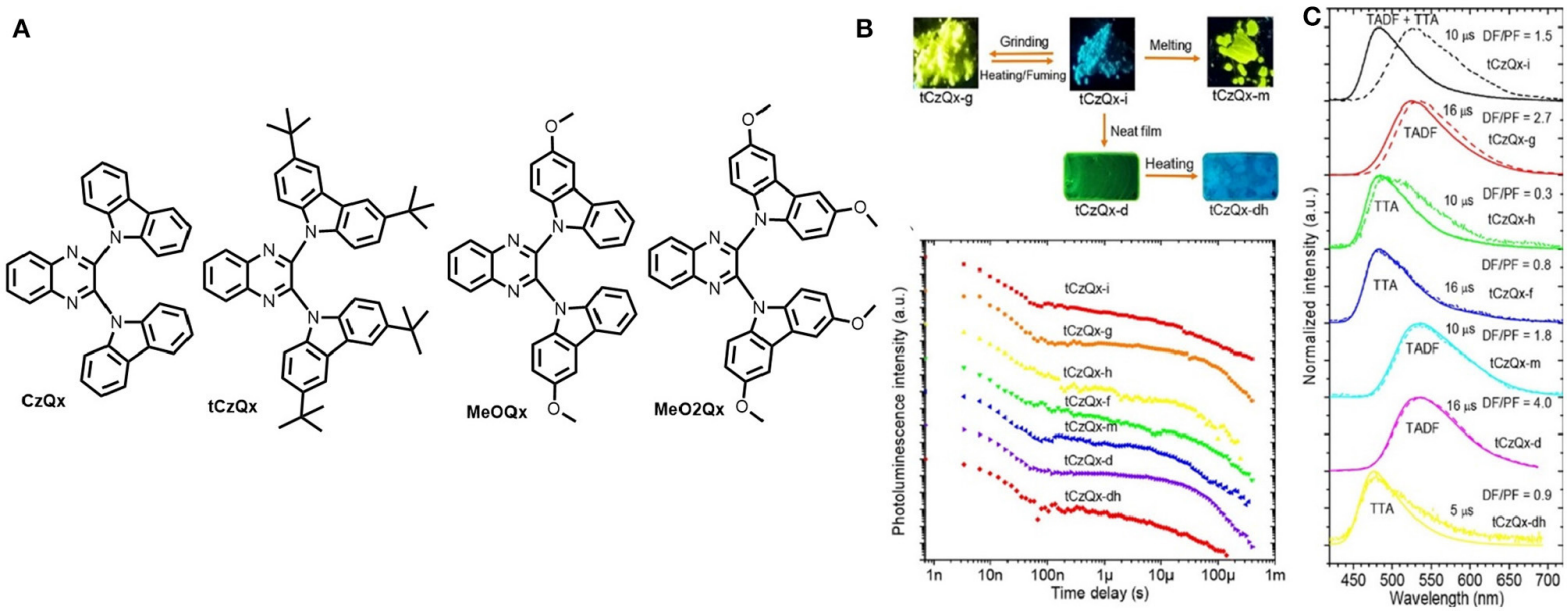
**(A)** TADF chemical structure of D-A-D triads. **(B)** Images of tCzQx at different forms (top-left); Distinct PL-decay plots of different states of tCzQx (bottom-left). **(C)** Time- resolved PL- spectra of the compound (solid lines: prompt fluorescence, dashed lines: delayed fluorescence). Reproduced with permission from Pashazadeh et al. (2018b). Copyright © American Chemical Society.

A series of four new D-A-D type TADF-MCL luminogens with reversible turn-on/off TADF properties in the solid state have been reported by the group of J. V. Grazulevicius (Pashazadeh et al., 2018b). In their study, the significance of the synthetic procedure is that it involves a smaller number of synthetic steps, which are catalyst-free and which have high product yields. Moreover, an electron-rich donor, 3-methoxy-9H carbazole, was used to examine the effect of methoxy group substitution on the carbazole units (Figure 7A). Notably, the rate of RISC (k_rISC_) is increased by increasing the donor strength from CzQx to MeO2Qx. The different colors exhibited by the molecule tCzQx were in response to different external stimuli (e.g., grinding, fuming, heating, melting) as shown in Figure 7B. Unlike previous reports, MCL materials can be well-aligned, usually by phase transition between crystalline and amorphous state, where delayed emission was not scrutinized precisely; however, in this work, the TADF property of each crystalline, microcrystalline, and amorphous states of the molecules was monitored properly (Figure 7B). Emission of tCzQx-dh was found to be quenched in the crystalline form where, amorphous and film tCzQx material showed perfect TADF property. Due to TTA and the increase in singlet CT energy, the singlet-triplet energy gap turns to high, thereby, quenching TADF in the crystalline state. A summary of theoretical and photophysical characterizations for MeO2Qx is provided in Table 1. Furthermore, based on these emitting materials, solution processed OLEDs were fabricated, which gave impressive EQE up to 10.9% (Table 2).

**Figure 8:**
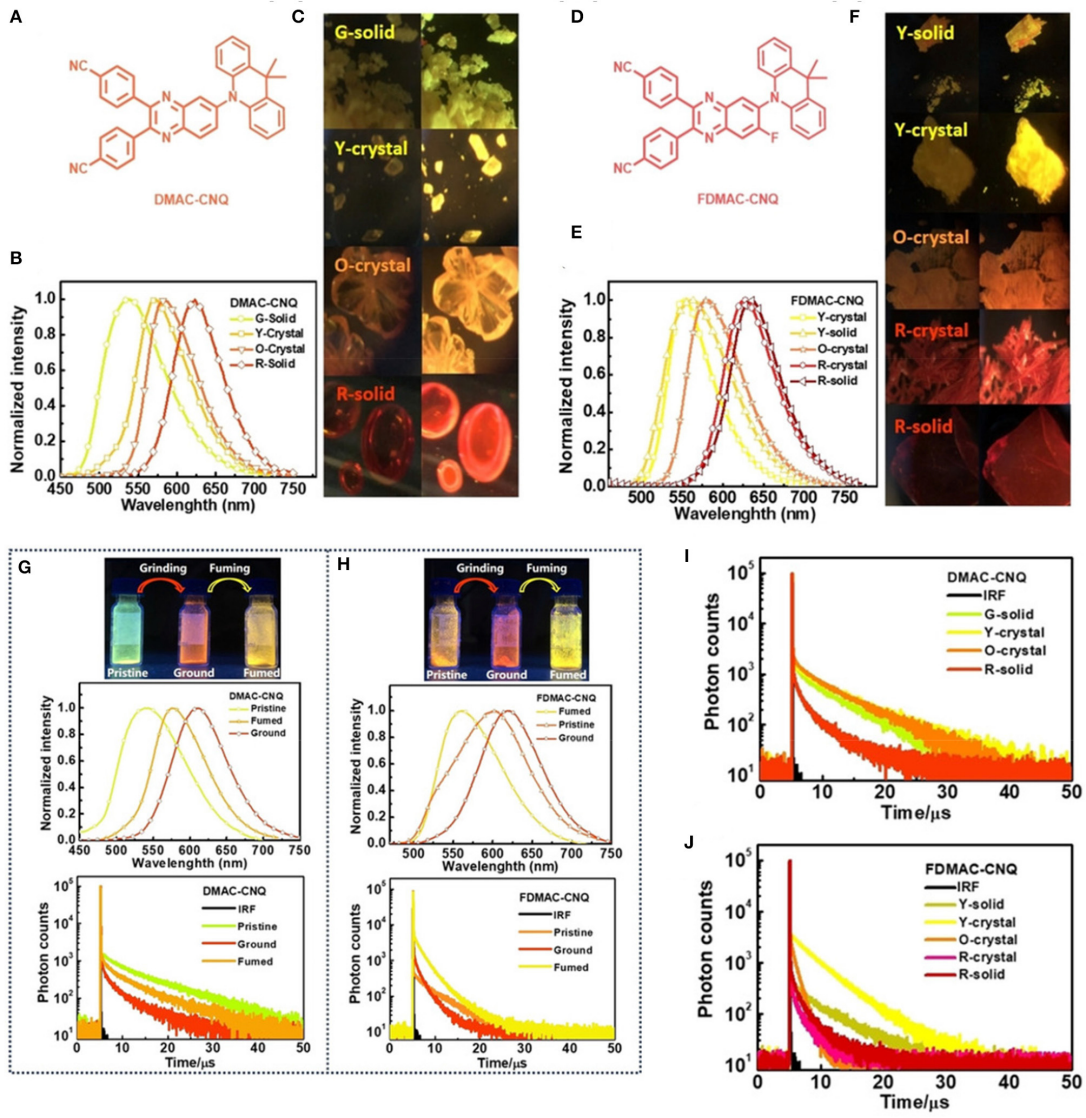
**(A–F)** Chemical structures, Photographs, normalized emission spectra of obtained polymorphic solids of DMAC-CNQ and FDMAC-CNQ, respectively. **(G,H)** Photographs and normalized PL spectra of mechanochromism properties and **(I,J)** transient PL decay spectra of the two TADF-emitters. Reproduced with permission from Zheng et al. (2019a). Copyright © WILEY-VCH Verlag GmbH andamp; Co. KGaA, Weinheim.

In a very recent report, another exciting finding by Zheng et al. (2019a) demonstrated how molecular packing in condensed states regulated the TADF phenomenon intensely by introducing two multifunctional orange-red AIE-TADF emitters, DMAC-CNQ and FDMAC-CNQ, respectively (shown in Figure 8). A deep insight established the relationship between multi-conformational aggregation states and TADF features, in terms of emission wavelengths, lifetimes, and PLQYs. In addition, a detailed crystal structure analysis revealed a structure-property relationship with diverse molecular stacking modes obtained from polymorphs, which determined the impressive TADF characteristic at the aggregated state. Both the emitters exhibited multicolor-MCL, and through grinding, greenish yellow color fluorescence changed red, and after fuming with CH_2_Cl_2_ vapors, the emission turned yellow (photophysical and theoretical results are summarize Table 1 for the emitters). This emission switching behavior also effects the TADF characteristics in powder form. Overall, this work concluded that the “aggregation state affects the TADF emissive feature.”

**Figure 9:**
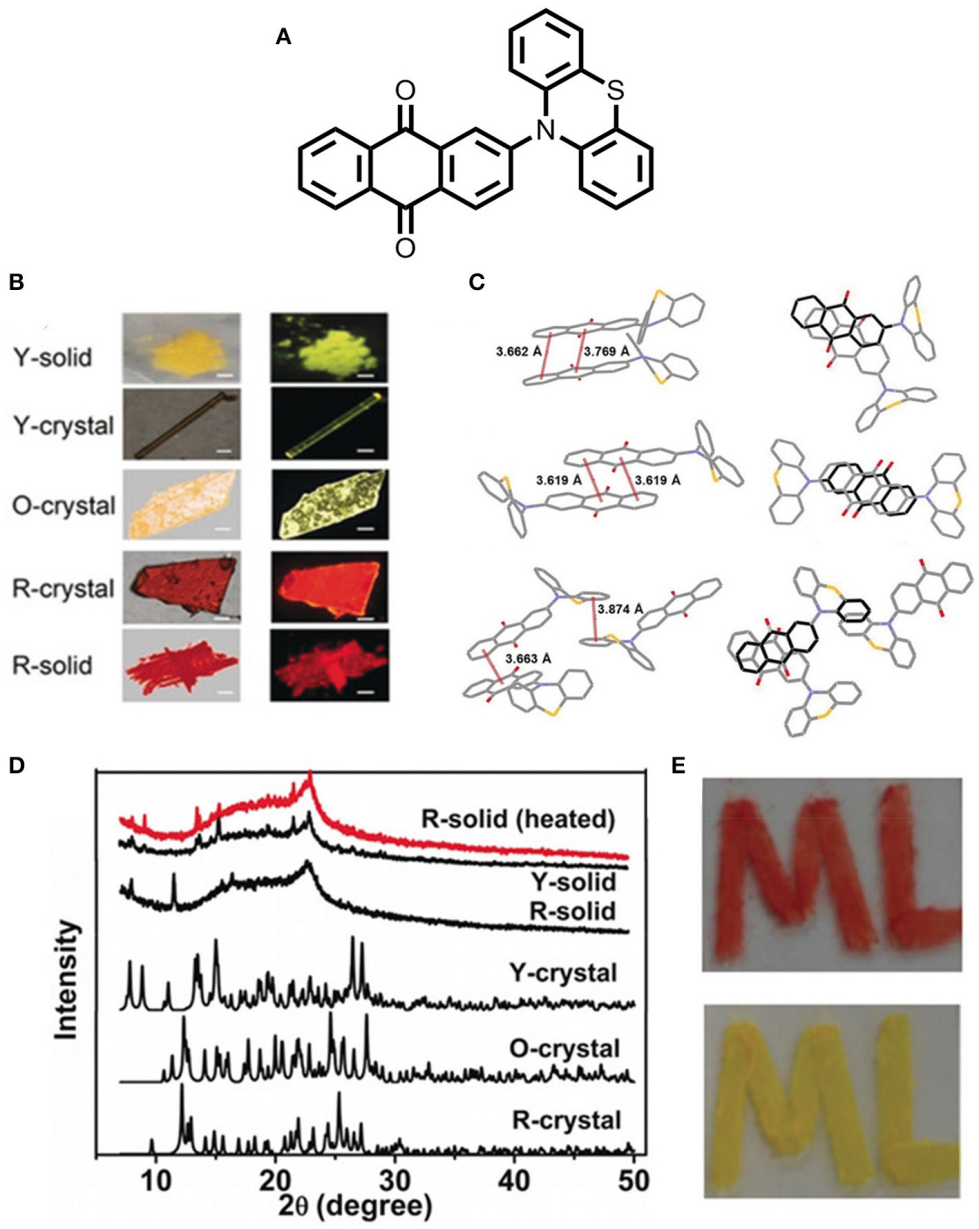
**(A)** Structure of D-A type PTZ-AQ molecule. **(B)** Photographs taken at different solid states (under ambient light-left; under UV irradiation- right; scale bar: 200 μm). **(C)** Intermolecular non-covalent interactions (along the b axis) of Y-crystal, O-crystal and R-crystal, respectively. **(D)** P-XRD pattern of different solid states of PTZ-AQ. **(E)** Image of the drown “ML” shape by the R-solid of PTZ-AQ under daylight (top); the image of shape “ML” (up) under heating condition at 150°C for 30 s in daylight (bottom). Reproduced with permission from Huang et al., 2018. Copyright © WILEY-VCH Verlag GmbH andamp; Co. KGaA, Weinheim.

Huang et al. (2018) reported an exciting multi-assets anthraquinone derivative, 2-(phenothiazine-10-yl)-anthraquinone (PTZ-AQ), which shows polymorphism, multicolor emission, MCL, AIE, and TADF properties in a single molecular platform (shown in Figure 9). In particular, the molecule consists of a planar acceptor moiety (AQ) and a non-planar donor moiety (PTZ), caused by a huge twist within the structure, which endows the better separation of the HOMO and LUMO, and further leads to a very small ΔE_ST_ value. Additionally, the non-rigid structure of the molecule is very much susceptible to the modulation of the morphology and molecular interactions, thereby, their respective photophysical properties with different aggregation states (Y-solid, R-solid, Y-crystal, O-crystal, and R-crystal). It is worth noting that the distinct crystal structure and different intermolecular interactions of the polymorphs are responsible for the attributed phenomenon, which cover tunable green to a deep red emission. The aggregation state emission has a substantial impact on the TADF properties of a molecule by regulating the molecular assembling modes. Unlike other polymorphs of PTZ-AQ, R-crystal displays efficient TADF characteristics owing to the very small ΔE_ST_ value of 0.01eV and shows excellent TADF PLQY up to 84.8%. Moreover, this work opens up a new approach for further research on the multifunctional organic emitters (detailed theoretical and photophysical data deliberated in Table 1).

**Figure 10:**
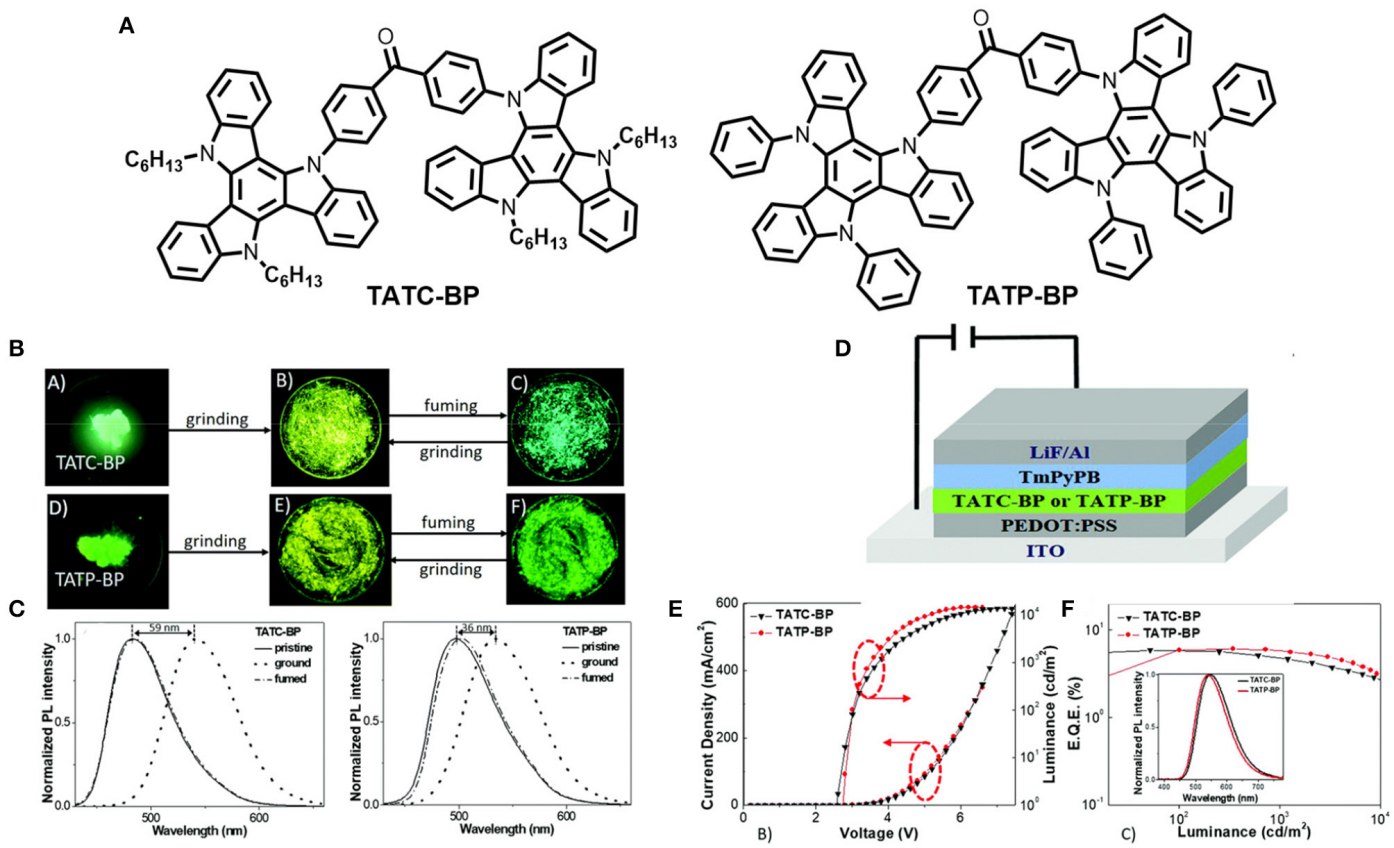
**(A)** Molecular structures of TATC-BP and TATP-BP **(B)** Luminescence images (under 365 UV-lamp) of the pristine crystalline, ground, and vapor fumed powders of the emitters. **(C)** Normalized MCL PL spectra of the emitters. Device configuration. **(D)** J-V-L graph **(E)** EQE and EL characteristics. Inset: EL spectra of the OLED devices at 1,000 cd ${\text{m-}}_{.}^{2}$ Reproduced with permission from Chen et al. (2018). Copyright ©The Royal Society of Chemistry.

Two new solution-processable triazatruxene-based small molecules, Bis(4-(10,15-dihexyl-10,15-dihydro-5H-diindolo[3,2-a:30,20-c]carbazol-5-yl)phenyl) methanone (TATC-BP) and bis(4-(10, 15-diphenyl-10,15-dihydro-5H-diindolo[3,2-a:30,20-c]carbazol-5-yl)phenyl) methanone (TATP-BP), were designed and synthesized by Chen et al. (2018). The molecules consist of two triazatruxene (electron donor) moieties linked through a benzophenone unit (electron acceptor), exhibiting twisted D-A-D conformation and comprising TADF and AIE along with MCL properties (shown in Figure 10). TATC-BP contains hexyl substituents on the TAT unit, whereas TATP-BP had phenyl substituents, and the different AIE and MCL behaviors of the molecules were controlled by the different substituents on the TAT unit (detailed computational and photophysical results summarize below Table 1). Due to the sterically less hindered and flexible alkyl substituents in TATC-BP compared to bulkier and rigid phenyl substituents in TATP-BP, the former crystal has a more compact packing, stronger intermolecular interaction with a smaller molecular reorganization energy, and emission is more shifted toward the blue region relative to the TATP-BP crystal. Furthermore, solution processed OLEDs were fabricated using these luminescent materials as an emissive layer and a maximum EQE of 5.9% (non-doped) and 15.9% (with 30 wt % doping) were achieved for the TATC-BP based device, whereas the TATP-BP based device showed a maximum EQE of 6.0% (non-doped) and 15.4% (30 wt% doping). Fascinatingly, the emitters achieved a low efficiency roll-off ratio. TATP-BP showed a much lower efficiency roll-off relative to TATC-BP, which indicates the effect of stiff and bulky phenyl groups.

**Figure 11:**
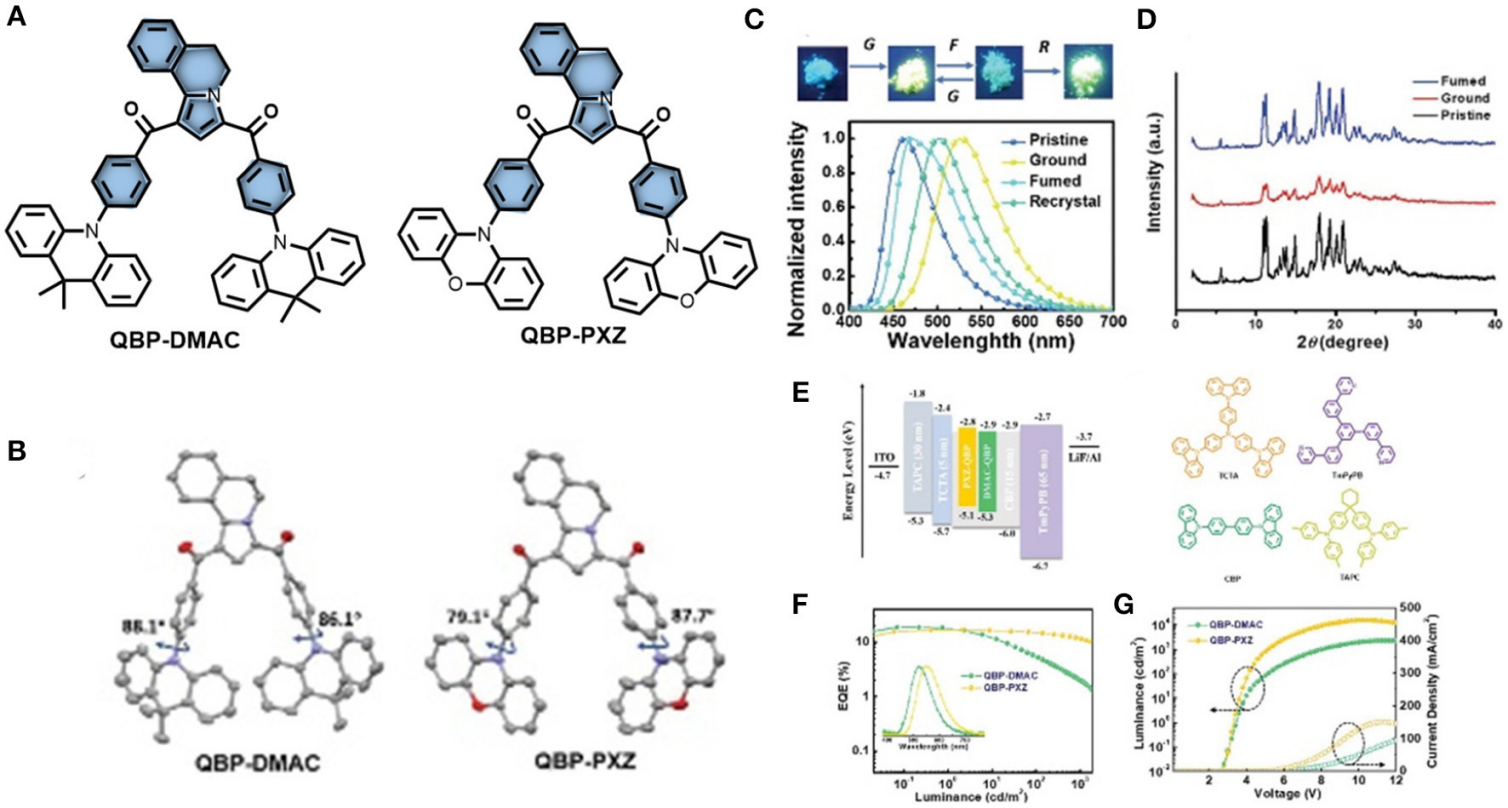
**(A)** Chemical structure of QBP-PXZ and QBP-DMAC **(B)** molecular configurations in single crystal XRD (hydrogen excluded). **(C)** PL spectra and MCL images of QBP-DMAC in (under 365 nm UV light) **(D)** PXRD patterns of QBP-DMAC. **(E)** Energy level diagram, device and material structures **(F)** luminance vs. EQE plots. Inset: Normalized EL spectra. **(G)** Luminance–voltage–current density plots. Reproduced with permission from Zheng et al. (2019b). Copyright ©WILEY-VCH Verlag GmbH andamp; Co. KGaA, Weinheim.

Zheng et al. (2019b) designed and synthesized two multifunctional D-A-D type emissive molecules (QBP-DMAC and QBP-PXZ) comprising a novel accepting unit (5,6-dihydropyrrolo[2,1-a]isoquinoline-1,3-diyl) bis(phenylmethanone) (QBP). Both emitters exhibit TADF and AIE properties with very low ΔE_ST_ values, realized by their highly twisted conformations with dihedral angles (between donor and acceptor) of 87.7 and 79.1° for QBP-PXZ, and 86.1 and 88.1° for QBP-DMAC; including QBP-DMAC which shows MCL characteristics (shown in Figure 11). Thermal up conversion of triplet excitons was further confirmed by the temperature dependent (77 K to 300 K) transient PL study. The PL intensity of the delayed component increased gradually with an increase in temperature. The underlying mechanism for this multi-color-MCL behavior has been further analyzed by a PL study and powder X-ray diffraction (PXRD) measurements, which revealing that the reversible phase transitions between amorphous and crystalline states, during and after the application of external mechanical force, are responsible for MCL. The careful crystal analysis of QBP-DMAC reveals that it has a loose molecular packing mode and weak π-π interactions between DMAC units, which are responsible for the underlying mechanism for its multicolor tunable MCL property. The photo physical and computational data for QBP-DMAC are given in Table 1. Based on these emitting materials, OLEDs were fabricated and a maximum EQE of 18.8% was achieved for QBP-DMAC-based OLEDs, which is among the highest efficiencies of TADF-AIE-MCL active organic emitters.

**Figure 12:**
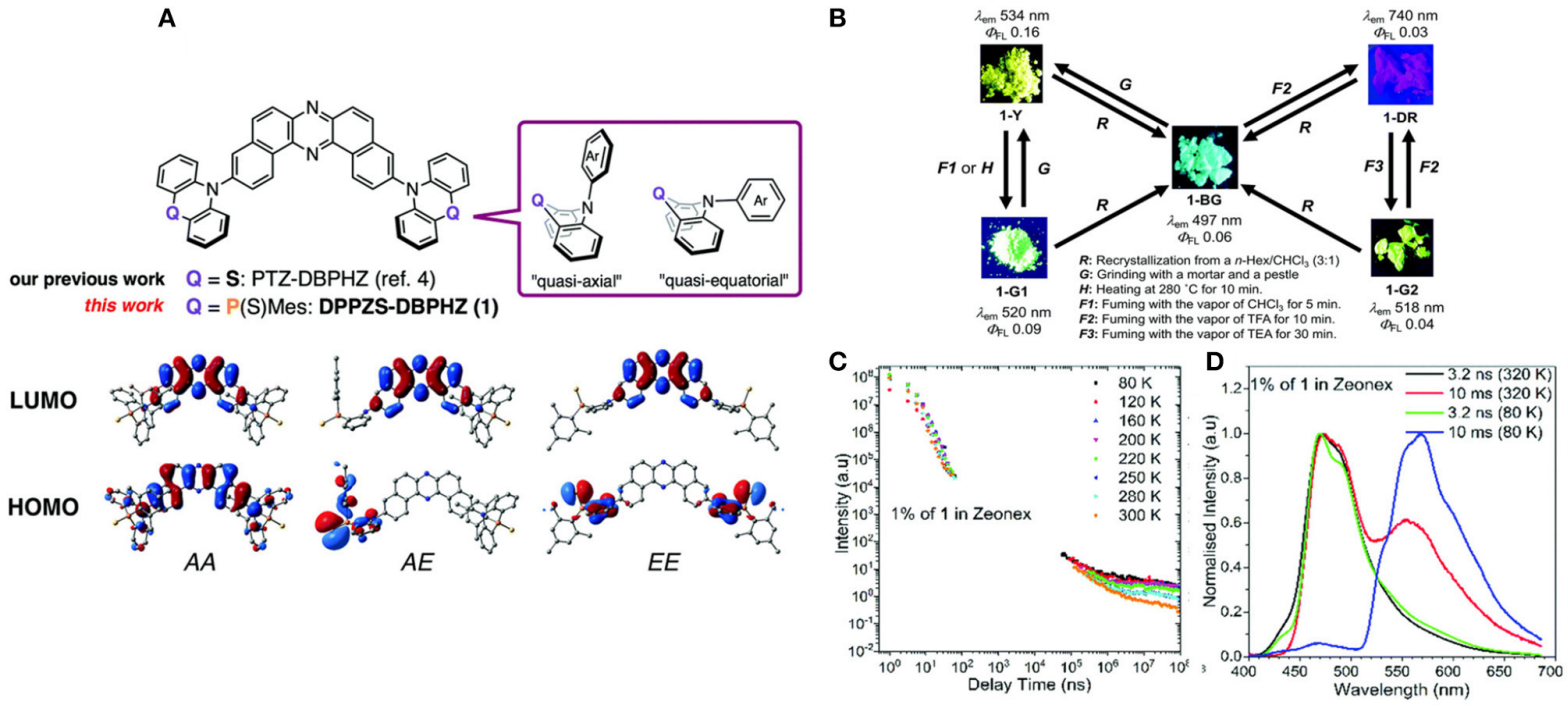
**(A)** Chemical structures of investigated molecules and the frontier orbitals of the conformers of 1. **(B)** Summary of the Mechanoluminescence property of 1 **(C)** Emission intensity at various temperatures of DPPZS-DBPHZ against delay time **(D)** PL spectra (Normalized) of 1 at various temperatures. Reproduced with permission from Takeda et al. (2018). Copyright © The Royal Society of Chemistry.

Multi-properties of a novel π-conjugated remarkably twisted D-A-D were investigated in purely organic triad DPPZS–DBPHZ, by Takeda et al. (2018), containing moderately-electron-donating and conformationally flexible units, where dibenzo[a, j]phenazine (DBPHZ) has been chosen as an acceptor and dihydrophenophosphanizine sulfide (DPPZS) as donors (shown in Figure 12). Fascinatingly, the compound exhibited multi-color-changing mechanochromic luminescence (MCL) assisted by conformational interconversion in the molecule, including TADF and unexpected RTP characteristics in presence of the host matrix (ZEONEX®). The delayed emission (DF) time constants decreased gradually, with an increase in the temperature. Furthermore, the molecule undergoes significant acid/base-responsive emission tuning between the visible and NIR region. Their theoretical and photo physical features are included in Table 1.

**Figure 13:**
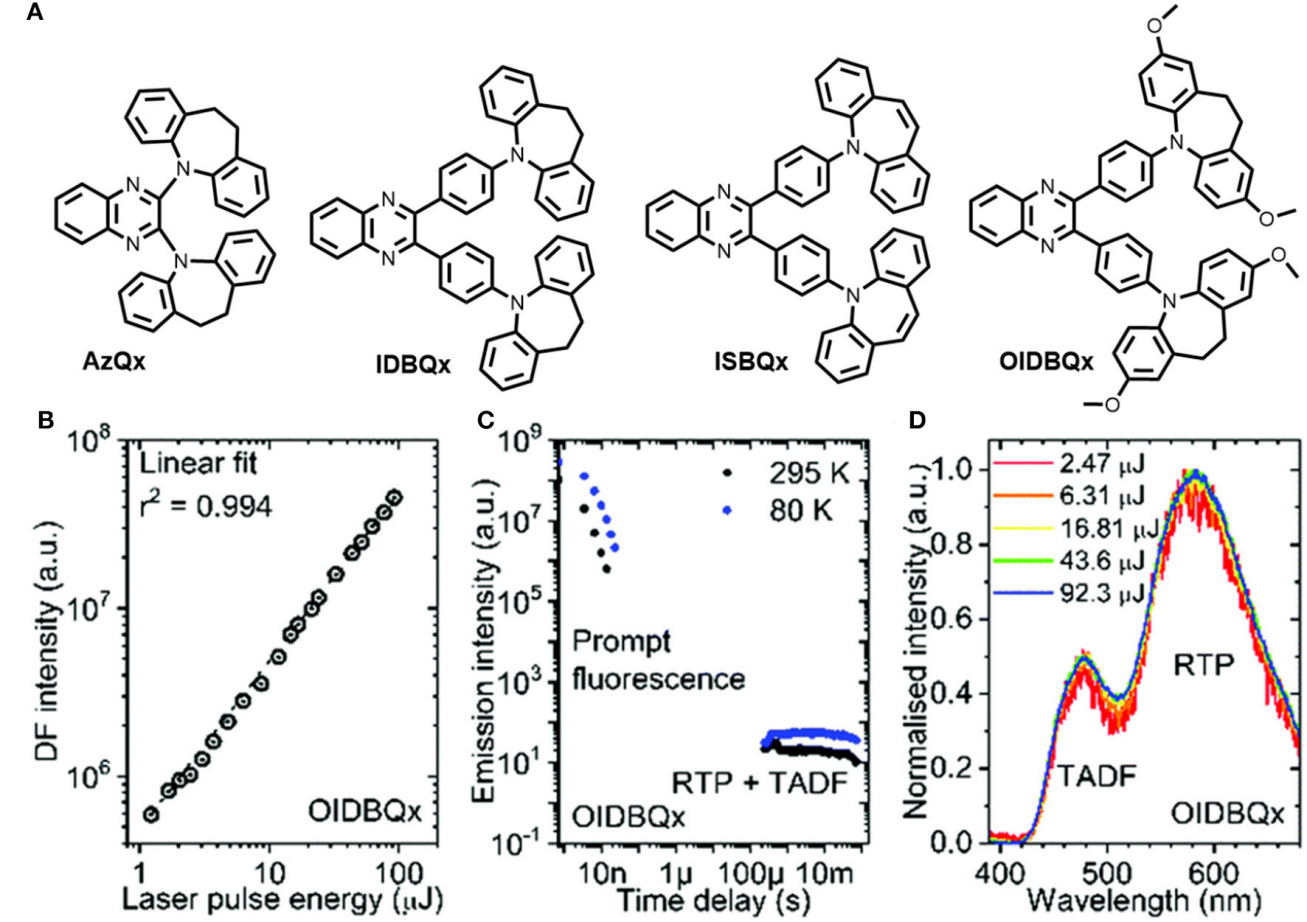
**(A)** Structures of AzQx, IDBQz, ISBQx, OIDBQx. **(B)** DF intensity vs. laser pulse energy plot of OIDBQx (at 295 K); **(C)** PL decay transients of OIDBQx at different temperature; **(D)** DF and RTP spectra (at 295 K) with different excitation pulse energy. Reproduced with permission from Pashazadeh et al. (2018a). Copyright © The Royal Society of Chemistry.

Tashazadeh et al. reported four new derivatives of quinoxaline-containing iminodibenzyl and iminostilbene moieties with TADF, RTP, and MCL properties (shown in Figure 13) (Pashazadeh et al., 2018a). To perceive RTP and TADF simultaneously, a modest singlet–triplet energy splitting is required to tune the RISC rate, to directly harvest all the triplet excitons (phosphorescence). Despite having a large singlet-triplet energy gap, room temperature delayed fluorescence and phosphorescence properties were observed for these luminophores (0.49–0.52). They have developed a quinoxaline-based acceptor of new luminogens. Notably, the emitters IDBQx, ISBQx, and OIDBQx, connected through a phenylene group between the acceptor and the donor units, exhibited orange RTP and blue TADF emission. This is a clear indication of non-aromatic twisted conformation. IDB not only induces strong spin–orbit coupling, but also minimizes the non-radiative decay. Remarkably, TADF or RTP were not observed for ISBQx, and only exhibited prompt fluorescence. Therefore, the non-radiative process was supported by the boat shape structure of ISB (7-membered ring in ISBQx), which triggered the quenching of triplet excitons. The methoxy group containing iminodibenzyl derivative exhibited MCL behavior due to the increased dipole moment. The introduction of a phenyl spacer between the donor and acceptor units increased the RTP contribution and the non-radiative decay was greatly suppressed by the twisted iminodibenzyl donor and the presence of methoxy groups. Table 1 includes the photo physical features of OIDBQx.

**Figure 14:**
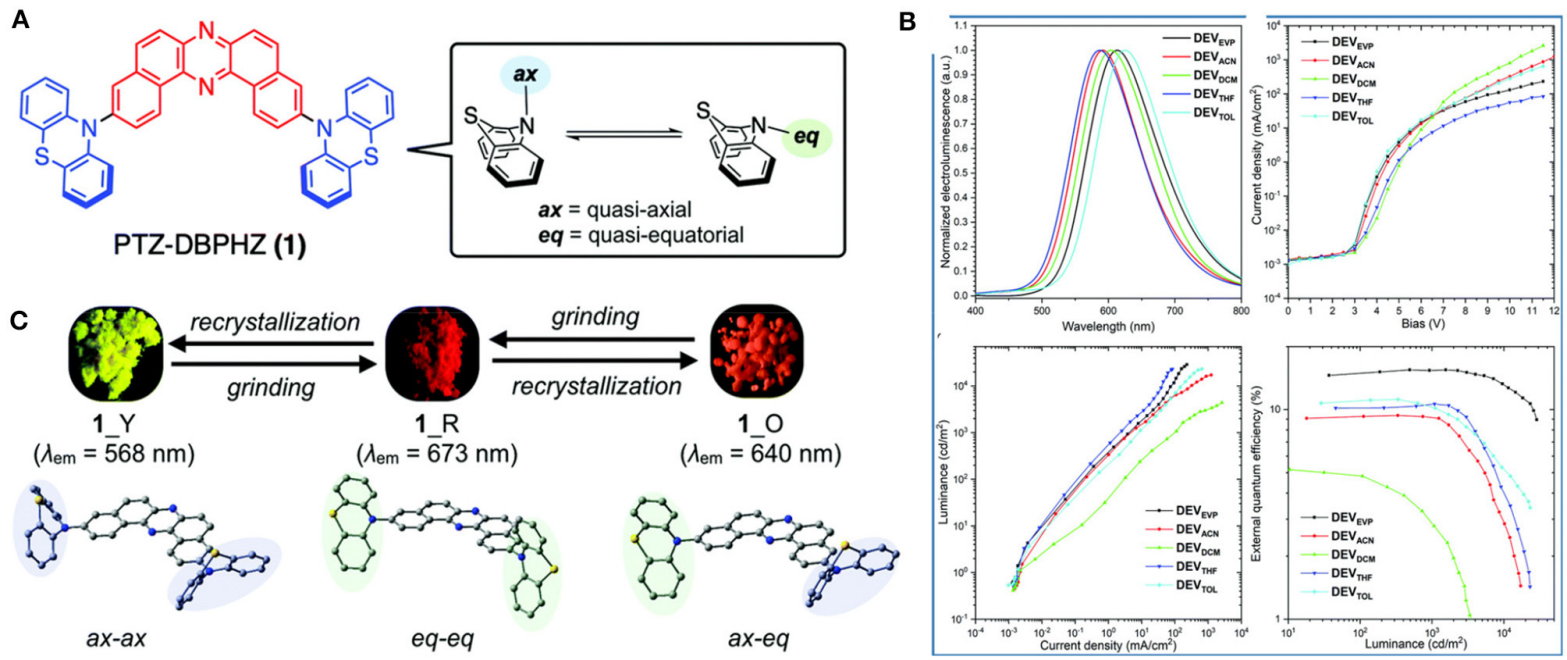
**(A)** Chemical structure of 1 with respective conformers **(B)** A comparison of characteristics of OLED devices and EL spectra **(C)** MCL properties in various conformers. Reproduced with permission from Data et al. (2019). Copyright © The Royal Society of Chemistry.

A multi-color-changing D-A-D type MCL-TADF-RTP material PTZ-DBPHZ was developed by Data et al. (2019) and it has been disclosed that the photophysical properties and multicolor MCL can be tuned using specific conformer-enriched [i.e., 1_Y: quasi axial–quasi axial (ax–ax), 1_R: quasi equatorial–quasi equatorial (eq–eq), and 1_O: quasi axial–quasi equatorial (ax–eq)] solids (shown in Figure 14). TADF and RTP characteristics were boosted depending on the dominant conformers of the molecule and were further employed in solution processed OLED devices, which gave excellent performance parameters. The data resulting from the photo physical studies for 1_R are included in Table 1.

**Figure 15:**
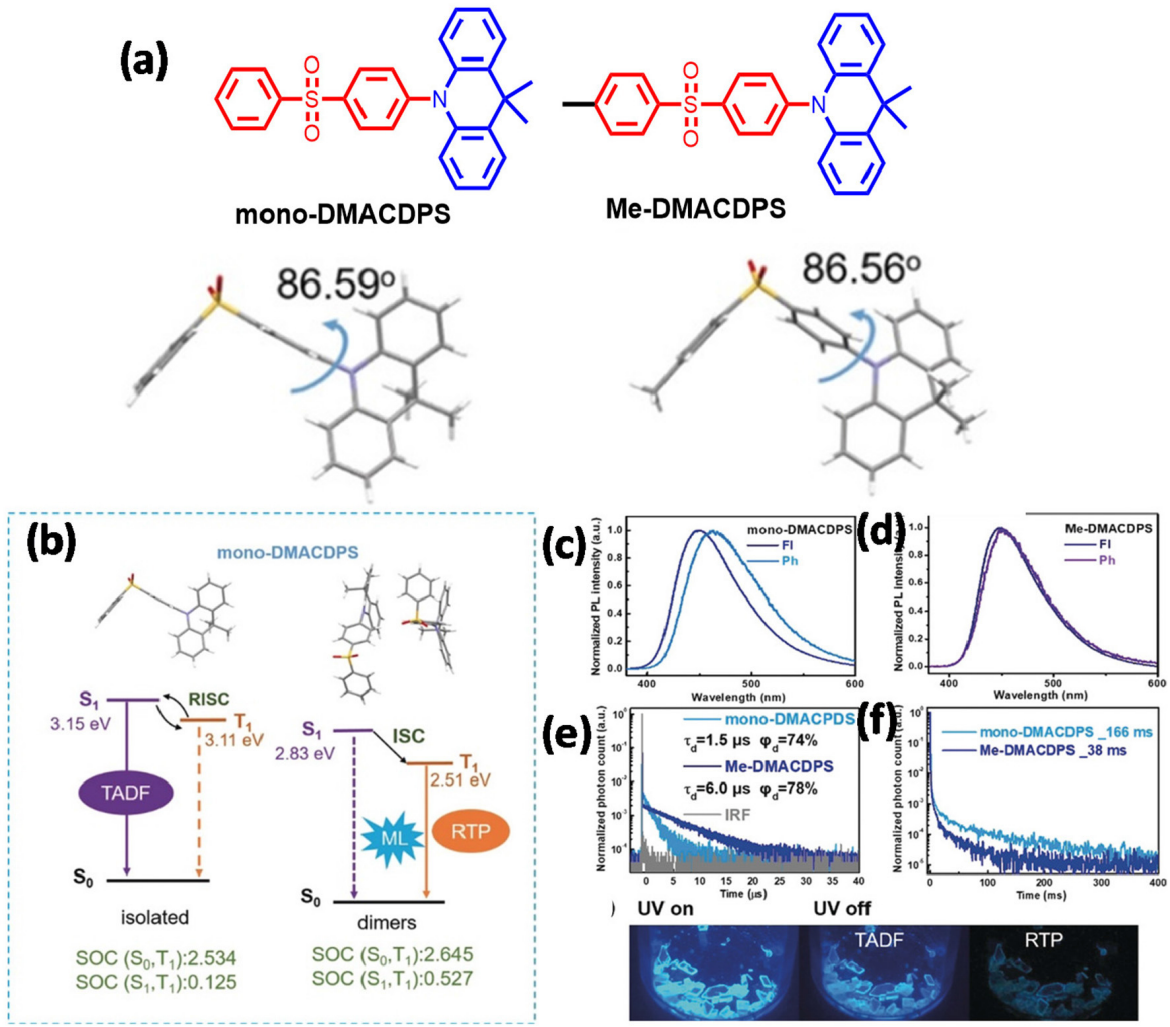
**(a)** Configurations and XRD structures of the molecules. **(b)** Energy level diagrams (of the monomer and dimer of mono-DMACDPS). **(c,d)** PL spectra of studied molecules (at 300 K). **(e,f)** The TRPL spectra of studied molecules. Bottom Crystal images of mono-DMACDPS (UV lamp—on and off). Reproduced with permission from Zhan et al. (2019). Copyright ©WILEY-VCH Verlag GmbH andamp; Co. KGaA, Weinheim.

Zhan et al. (2019) reported the first example of multifunctional organic material with simultaneous MCL, AIE, RTP, and TADF properties (shown in Figure 15). Two blue emitters of mono-DMACDPS and Me-DMACDPS were designed and developed, where diphenyl sulfone acts as an acceptor and 9,9-dimethyl-9,10-dihydroacridine (DMAC) acts as a donor. DMAC is a conventionally used TADF emitter and possibly assists for AIE, whereas diphenyl sulfone is a commonly used acceptor comprising manifold modification sites. The emitters displayed typical TADF and AIE properties in the solution state, while RTP and TADF characteristics were displayed in their crystalline form. A methyl group was introduced to modulate the MCL property *via* regulating intermolecular interactions and packing mode by the steric hindrance of the methyl group, and Me-DMACDPS was found to be MCL inactive, whereas mono-DMACDPS was found to be exhibiting a distinct MCL property. Careful analysis of the crystal structure disclosed that mono-DMACDPS exhibit a large dipole moment and tight packing mode due to multiple intermolecular interactions that endow them with strong MCL (Table 1).

### Single Emitting White-TADF-MCL

Single molecule based white-light emitting TADF emitters have long-standing demand in solid-state lighting, display, and OLED applications due to their high triplet energy and full width at half maximum (FWHM). In addition, very few white-TADF emitters inherently manifest MCL behavior which are strongly governed by weak non-covalent interactions and constructed by a dual color emissive entity in a single molecular platform, enabling the transfer of full energy either to counter the green and red fluorescent emitter or by the complementary yellow emitter. Hence, a smart design and effective non-covalent interactions are prerequisites to realizing TADF-white light in the accompanying tunable emission.

**Figure 16:**
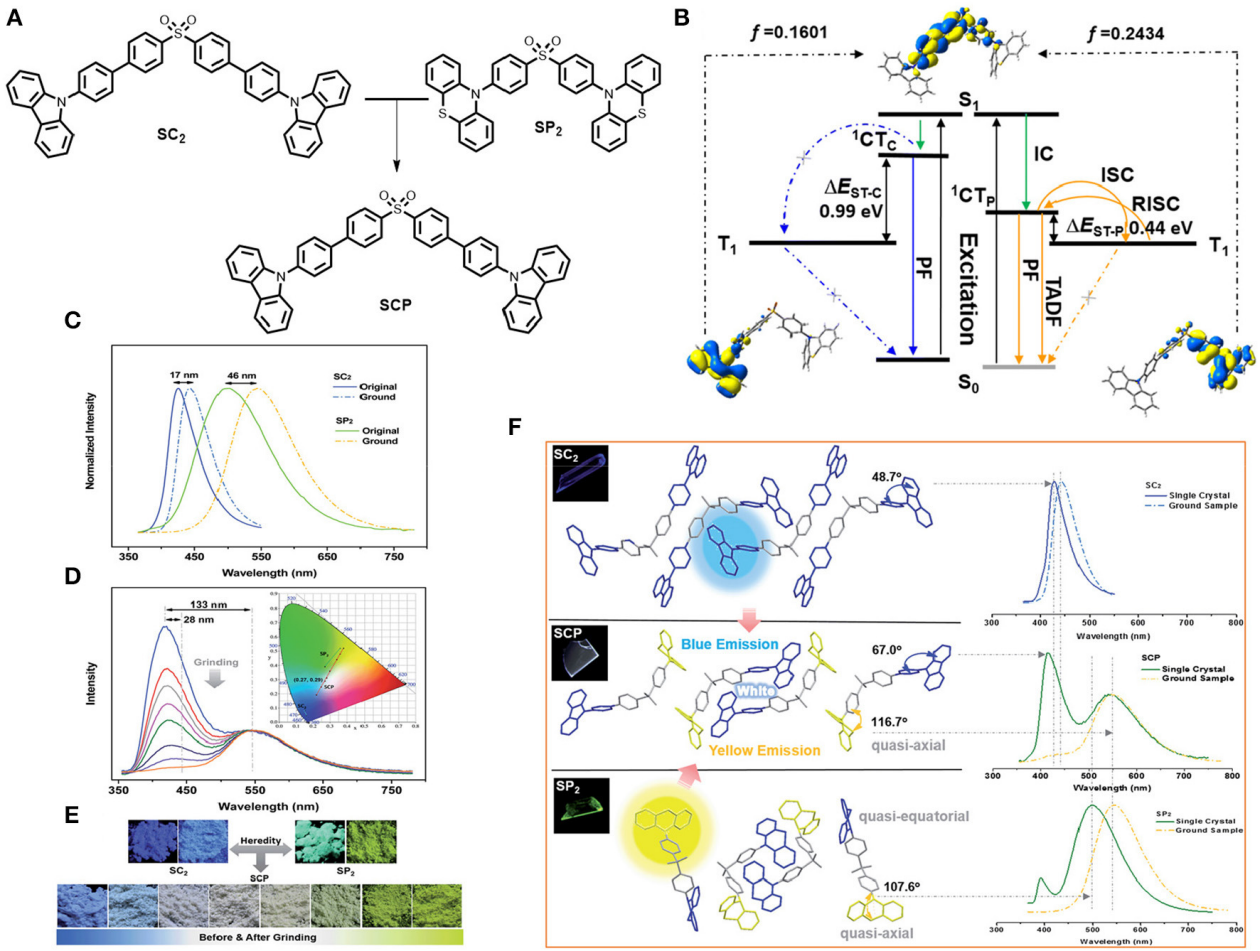
**(A)** Molecular configuration of the compounds. **(B)** Decays associated with corresponding electronic transitions in SCP. **(C)** PL study of the parent molecules in solid-powder form. **(D)** Mechanochromism in SCP molecule. **(E)** Compound images under the UV-irradiation of 365 nm. **(F)** Single crystal analysis and crystalline molecular packing of the compounds along with corresponding photo luminence spectra of the single crystals and normal compounds. Insets: fluorescence images (365 nm UV-excitation). Reproduced with permission from Xu et al. (2016). Copyright © The Royal Society of Chemistry.

Xu et al. (2016) established the strategy of molecular heredity to achieve high contrast linearly tunable mechanochromism with white light emission [CIE value: (0.27, 0.29)] from a single compound, and established the origin of the compound's dual emission (shown in Figure 16). The two parent molecules, SC2 and SP2, are symmetrical by means of donor, where SC2 exhibited deep blue emission and SP2 yields impressive greenish-yellow TADF emission. Interestingly, their offspring is an asymmetric molecule, namely SCP, which thus inherited both deep blue and greenish-yellow colors that combine to generate white light emission along with TADF by means of molecular heredity, enabling a potential candidate for multi-responsive and efficient white-light emitting photoelectric devices. The dual-emission behavior of SCP can be dispensed to two independent emissions of the excited charge transfer states of the carbazole and phenothiazine units, respectively. Moreover, the underlying mechanism of MCL for SCP driven by the mechanical strength, associates with the conformational planarization of the phenylcarbazole unit along with the stronger energy transfer from the blue band to the yellow light emission band.

**Figure 17:**
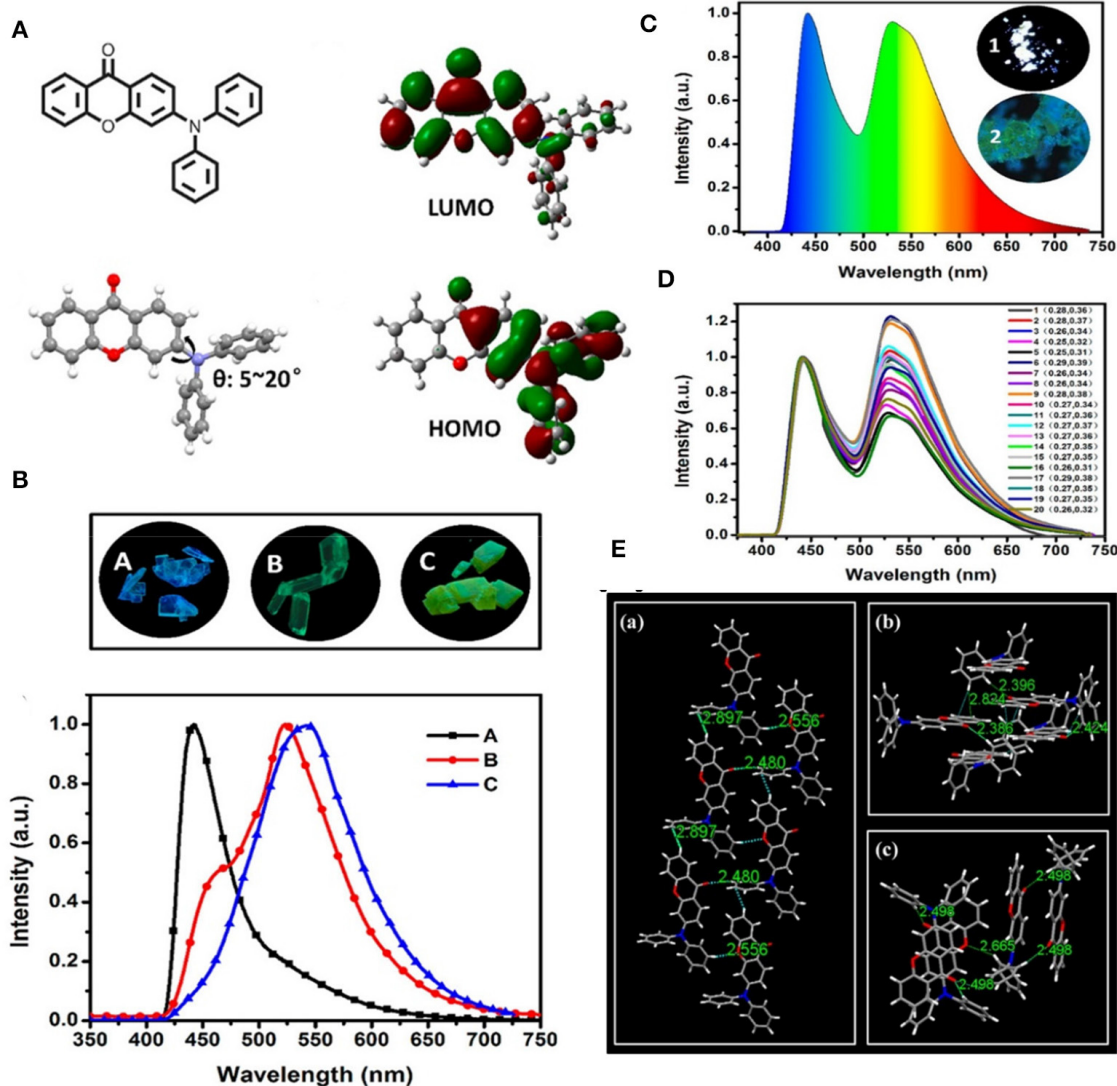
**(A)** Chemical structure of 3-DPH-XO (left side-top) followed by single crystal structure (left side-bottom) and theoretical spatial HOMO-LUMO distribution. **(B)** Photographs of the polymorphs with normalized fluorescence spectra (bottom) recorded under 365 nm UV excitation. **(C)** Result of 20 combined emission spectra of powdered-3-DPH-XO under 365 nm UV irradiation; inset: (1 and 2) fluorescence microscopy image of the powder. **(D)** Respective color-coordinates (CIE 1931) of 20 random emission spectra of solid- 3-DPH-XO under 365 nm UV excitation. **(E)** Intermolecular interactions and overlaps in A (a), B (b), and C (c) crystals. Reproduced with permission from Zhang et al. (2017). Copyright © American Chemical Society.

An efficient and simple D-A approach to achieve white light from organic solids with a polymorph dependent TADF property has been developed by Ban et al. (2017). A purely organic molecule of 3-(diphenylamino)-9H-xanthen-9-one (3-DPH-XO) has been reported, which was found to display bright white light emission with the CIE value (0.27, 0.35) in its solid state, facilitated by spontaneous formation of polymorphs with distinguished intermolecular non-covalent interaction features that determined the different emission colors, and which was further demonstrated by single crystal studies of the compound (shown in Figure 17). Three unprecedented different 3-DPH-XO-based single crystals were achieved with different supramolecular structures and emission properties and two of the polymorphs with acceptor-acceptor stacking revealed TADF characteristics (Table 1). This indicates that appropriate non-covalent interactions like π- π stacking, C-H-π interaction, and hydrogen bonding could offer a promising TADF feature by influencing the reverse intersystem crossing (RISC) process.

## Conclusion and Outlook

In summary, the rapid development of TADF research has resulted in a new dimension of luminescence features, by building up the substantial information into new material designs, exciting and unique key functional behaviors, and essentially providing a mechanistic understanding of TADF processes. However, this review has attempted to concentrate on the recent progress made in the development of multifunctional metal free organic emitters, focusing on aggregate state emissions, TADF, and other multi-functionalities such as ML, MCL, ECL, and white light emission properties in a single molecular platform. Furthermore, a brief overview was provided on the structure-property relationship between self-assembled solid state and excited state dynamics in order to explore the opportunities for the rational design of multiple emission functions into single organic molecules and the further implementation into lighting applications, which are still in their infancy. In addition, this review includes the “state of the art” simple OLED device fabrications protocol and emphasized impressive output by exploiting multi-function TADF materials. More importantly, these multifunctional emitters are considered to be promising candidates for solution processed economical device fabrications owing to their tunable condensed state emissions. We believe that the rapid developments taking place in this field is generating enthusiasm, motivating researchers to explore a deeper understanding and finding new advancements in this exciting area of research. We expect this review to provide a clear prospect and to attract researchers with diverse interests to these novel multi-functional materials, to devote themselves to the development of materials, methods, and applications in this interesting interdisciplinary topic.

## Author Contributions

DB planned and compiled the review article. RG compiled the text, figures, and references. KN compiled the text and references. PI initiated, planned, and compiled the review. All authors contributed to the article and approved the submitted version.

## Conflict of Interest

The authors declare that the research was conducted in the absence of any commercial or financial relationships that could be construed as a potential conflict of interest.
